# Bats Out of Africa: Disentangling the Systematic Position and Biogeography of Bats in Cabo Verde

**DOI:** 10.3390/genes11080877

**Published:** 2020-08-01

**Authors:** Ianna Borloti, Herculano Dinis, Raquel Vasconcelos

**Affiliations:** 1 CIBIO-InBIO, Centro de Investigação em Biodiversidade e Recursos Genéticos, Laboratório Associado, Universidade do Porto, Campus Agrário de Vairão, 4485-661 Vairão, Portugal; iannaborloti@gmail.com; 2 Departamento de Biologia, Faculdade de Ciências, Universidade do Porto, 4169-007 Porto, Portugal; 3 Parque Natural do Fogo, Direcção Nacional do Ambiente, 115 Chã d’Areia—Praia, Santiago, São Lourenço dos Orgãos CP 84, Cape Verde; pnfogo.segecol@gmail.com; 4 Associação Projecto Vitó, 8234 Xaguate, Cidade de São Filipe, Fogo 8220, Cabo Verde

**Keywords:** chiropters, distribution, *Hypsugo*, islands, genetics, *Miniopterus*, *Plecotus*, *Taphozous*

## Abstract

Cabo Verde Archipelago presents one of the largest knowledge gaps in the distribution and taxonomy of bats in the world. Old works indicated that there are five species classified as European taxa. We have conducted an integrative taxonomy to revise the systematic position and distribution of Cabo Verdean bats with molecular, morphological, and ecological data, to test their native or exotic origin, and infer possible colonization patterns based on fieldwork and museum samples. Results showed that Cabo Verde *Hypsugo* is closely related to those from the Canary Islands, in which the taxonomic status is under debate, presenting unique mitochondrial and nuclear haplotypes. We also expanded the distribution of *Taphozous nudiventris* for Fogo Island through pellets and acoustic identification, showed unique haplotypes for this species, and that *Miniopterus schreibersii* shared a haplotype with European, North African, and Western Asian specimens. The morphological and acoustic identification of Cabo Verdean specimens was challenging because of the lack of modern morphological descriptions and similarity of echolocation calls within the same genus. More studies are definitely needed to access the systematic of bat species in the archipelago, but this work is the first step for the establishment of conservation actions of the probable only native Cabo Verdean mammals.

## 1. Introduction

Biodiversity loss is one of the major environmental problems, threatening valuable ecosystem services [1]. In order to have an accurate picture of the biodiversity loss, accurate species inventories and their distribution are needed [2,3]. The assessment of global biodiversity is a challenge because most species were still not formally described and the geographical distribution of most species are poorly understood, usually referred to as Linnean and Wallacean shortfalls respectively [2,4,5]. Given the limited resources and urgency for biodiversity rescue, a rapid assessment method is required [6]. Phylogenetic analysis has a huge impact on the understanding of relationships among lineages, adding power and robustness to biodiversity assessment [5]. The number of bats described has been increasing dramatically since the beginning of the 20th century because of advances in DNA sequencing. Spectacular levels of cryptic diversity have been frequently reported in Europe [7,8,9,10,11,12,13].

In all habitats, acoustic sampling is a very effective methodology to survey and monitor bats [14]. They use a wide variety of species-specific signal types differing in frequency structure, duration, and sound pressure level that can be used for species identification [14,15]. Although traditional procedures will remain useful in many cases, taxonomy needs to be integrative with new approaches for species delimitation [16]. The combination of morphological measures, population genetics, ecological, and phylogeographic data provide to taxonomists a larger arsenal to better face the huge challenge of inventorying biodiversity [16,17].

Biodiversity is not homogeneously distributed; it varies widely throughout different regions. It is particularly high on oceanic islands. They are well-known centers of endemism and range-restricted species, hosting some unique lineages and some of the most threatened species [18,19]. In these localities, the Linnean and Wallacean shortfalls are even more profound because more scientific investments and effort are needed to elucidate cryptic diversity and species ranges [18,19]. Bats are a major component of mammalian biodiversity and are often the only native mammals in oceanic islands [20,21,22]. Nevertheless, bats are often underrepresented in conservation and management plans because of the knowledge gaps on population structure, distribution, and habitat requirements [23].

Very few studies and field surveys have focused on bats from Cabo Verde. The archipelago possesses one of the largest knowledge gaps in the distribution and taxonomy of those species [24]. Data published in the 1960s and 1980s indicate that there are five species in the archipelago, all considered with recent colonization and reduced distribution. Moreover, the majority are classified as European taxa. The species were identified as the naked-rumped tomb bat *Taphozous nudiventris* Cretzschmar 1830; the Savi’s pipistrelle *Hypsugo savii* Bonaparte 1837; the Kuhl’s pipistrelle *Pipistrellus kuhlii* (Kuhl 1817); the gray big-eared bat *Plecotus austriacus* (Fischer 1829); and the Schreiber’s bent-winged bat *Miniopterus schreibersii* (Kuhl 1817) [24,25,26,27,28,29,30]. There is also an observation of the African straw-colored fruit-bat *Eidolon helvum* (Kerr 1792), which is probably a vagrant specie and an indeterminate molossid species [24,29,31,32]. There is also a lack of information about how they reached the archipelago, if they have colonized the islands by passive transport, as boats, or if they have reached the islands by their own means.

The systematic status of the Cabo Verdean bats is still controversial, especially on those complexes of sympatric and cryptic species widespread throughout Europe and North Africa. One of those species is *H. savii,* which has a wide geographic distribution in the Palaearctic region. In the Macaronesia archipelagos, *H. savii* has been reported on La Palma, Tenerife, La Gomera, Gran Canaria, and El Hierro in the Canary Islands, and on Fogo, Brava, São Nicolau, São Vicente, and possibly Santo Antão in the Cabo Verde Islands [24,33]. The systematic and taxonomy of the genus is still far from being sufficiently resolved. The number of known lineages has been increased through molecular analyses (e.g., [9,10,33,34,35]). More recently, within mitochondrial DNA (mtDNA) markers cytochrome *b* (*cyt b*) and *NADH* dehydrogenase subunit 1 (*ND1*), three main lineages in Europe diverged by over 7% [9,13]. One lineage was found on southern Iberia populations that may be recognized at subspecific level, which is also widespread in Morocco, whereas another Iberian lineage that occurs as far north as Switzerland [9]. Both lineages appear in sympatry in the southern Iberian Peninsula [13]. The third lineage corresponds to *H. savii* from eastern Mediterranean [9]. Other authors, analyzing the mtDNA of populations from Italy and the rest of Western Palearctic, found three haplogroups of *H. savii*: one Italian that clusters along with haplotypes from Maghreb, another Southern Iberian, and another east European [10].

In 2003 it was found that the divergence of Canary Islands population compared with mainland Iberia was 6.3–7.2% for *cyt b* and 3.8–4.4% for 16S ribosomal RNA (rRNA) [33], the latter was also confirmed by other authors [36]. In 2007, it was suggested that the African lineage corresponded to *Hypsugo cf. darwinii* (Tomes 1859) because of a high sequence divergence (of 9.6%) in the mtDNA *ND1* gene from the Iberian *H. savii* [12]. The relationship between Macaronesia bats and this African mainland lineage needs to be assessed before naming the species. Despite this unclear taxonomic status, *H*. *cf*. *darwinii* clearly constitutes a distinct evolutionarily significant unit (ESU) [36]. In the Sardinia Island, two *Hypsugo* haplolineages occurs in sympatry. One lineage refers to *H. savii* (*sensu strictu*), which is grouped together with the European haplotypes and the other lineage refers to *H. cf. darwinii*, which connected to the North-African/ Canarian group of haplotypes, also occurring in Turkey [36]. The *H*. *cf*. *darwinii* also occurs in Tuscany, more specifically in Montecristo Island [37], and more recently it was reported that *Hypsugo* from the Maltese islands is also related to North-African *H. savii* lineage and the *H. savii* (*sensu strictu*) might also occurs in sympatry [38]. Those studies suggested the hypothesis that this taxon might also be present in other Mediterranean and Macaronesian islands.

Regarding the taxonomic status of *P. kuhlii*, there is also some debate. The species is a typical representative of the Mediterranean bat fauna. It is widespread in the Palearctic realm [39,40]. In Macaronesia, *P. kuhii* is recorded on Gran Canaria, Fuerteventura, and Lanzarote in Canary Islands [33] and on São Vicente, São Nicolau, Fogo, and Santiago in Cabo Verde [24]. According to more recent genetic studies, European population of *P. kuhlii* consists of two main lineages (western and eastern) diverged by 6.1% for cytochrome c oxidase I (*COI*) [41] and 1.9% for 16S rRNA markers [42]. The western/ southern lineage is widely distributed across the Mediterranean basin, Canary Islands, and in the Balkans and it is referred to as *P*. *k*. *kuhlii* (Kuhl 1817), while the eastern lineage is referred to as *P*. *k*. *lepidus* Blyth 1845 and it occurs in Poland, Ukraine, Russia, Caucasus, and Middle East [42]. In addition, some morphological differences were found between the two lineages [42]. The North African lineage of the western lineage was described as *P. deserti* Thomas 1902. However, molecular studies suggested that this lineage is likely a recent adaptation to arid environments rather than presenting a long independent evolutionary history [41,43].

It was suggested that *P. kuhlii* and *Pipistrellus madeirensis* (Dobson 1878) from Canary Islands form a monophyletic clade with respect to *P. kuhlii* from mainland Spain [33]. Thus, *P. kuhlii* appears to be paraphyletic species due to the nesting of *P. maderensis* within it, which is more closely related to the Kuhl’s bats from Canary Islands than those that are related to Kuhl’s bats from outside the archipelago [33]. Observations have been made in La Palma, indicating that *P. kuhlii* may be present in most Canary Islands, while *P. madeirensis* has only been detected on the western islands and Madeira Archipelago [33]. Results found by the same authors indicate that these two species diverged 3.7–4.7% in *cyt b* mtDNA and 2.0–2.8% in 16S rRNA [33]. However, some intermediate phenotypes have been detected in the islands where both species co-occur (sometimes in the same sites) suggesting the hypothesis that both lineages may interbreed [33].

The species *P*. *austriacus* is essentially restricted to the Palearctic [44]. The genus *Plecotus* has proved to be good at colonizing distant islands, as it occurs on several Mediterranean and Atlantic Islands (e.g., Balearic Islands, Sardinia, Corsica, and Sicily) [44]. In Cabo Verde, it was recorded on Maio and Santiago [24]. Historically, a number of taxa have been described in the genus *Plecotus* based on morphological measurements [45], but there is a lack of clear diagnostic characters. The taxonomic status and geographic distribution of some species is still unclear. Recent molecular studies have revealed four to five new species apart from *P. austriacus*, *Plecotus auritus* Linnaeus 1758 (from Western Europe eastwards at least to Russia), and *P*. *teneriffae* Barret-Hamilton 1907 (from the Canary Islands), some still under debate: *Plecotus macrobullaris* Kuzjakin 1965 (a senior synonym for *Plecotus alpinus* Kiefer and Veith 2002 and *Plecotus microdontus* Spitzenberger 2002 from Caucasus, Asia Minor, the Alps and the Balkans to the Pyrenees [46,47]); *Plecotus kolombatovici* Dulic 1980 (originally described as subspecies of *P. austriacus*) from the Balkans and Italy [8,47,48,49,50]; *P*. *sardus* Mucedda, Kiefer, Pidinchedda and Veith 2002 endemic taxon from Sardinia Island [51]; an undescribed species occurring in Morocco, and the recent *P. gaisleri* Benda, Kiefer, Hanák, and Veith 2004 occurring in Maghreb and in the Italian Pantelleria Island [52]. Previous authors, analyzing mtDNA identified two major lineages, the “*auratus*” and “*austriacus*” group, each one is subdivided into two further subgroups [53]. Within the “*austriacus*” group, there is a clade attributed to *P. austriacus* (*sensu strictu*) from Central Europe to Southern Europe, including Madeira and the Balearic Islands [53]. The other subclade includes three very well differentiated lineages, the Canarian long-eared bat *P. teneriffae*, the undescribed Morrocan species, *P. gaisleri* [52], and *P. kolombatovici* [53]. It is unknown if the *Plecotus* that occurs in Cabo Verde is actually *P. austriacus* (*sensus strictu*) or *P. teneriffae*/ *P. kolombatovici*, or even a distinct lineage resulting from long period of isolation [54]. 

The *M. schreibersii* has a geographic distribution across Southwestern Europe, North and West Africa, Anatolia and the Middle East to Caucasus. The genus *Miniopterus* Bonaparte 1837 has a widespread distribution throughout most of Africa, Europe, Asia, New Guinea, Australia, and the Pacific [55]. The species of this genus are often difficult to identify morphologically given the lack of a discriminate characters [56]. Several classifications have been constantly reviewed and changed over the years. More recently, two major clades inside *M. schreibersii* complex were recognized, in which the distributions correspond to the Ethiopian-Palearctic and the Oriental-Australasian zoogeographical regions [57]. Each lineage splits further into more geographically structured branches. Some author suggested that *M. s. schreibersii* (Kuhl 1817) is a typical representative to Europe, North Africa, and Asia Minor and *M. s. pallidus* Thomas 1907 is present in the Asian part of Turkey [58]. Recently, the population structure of *M. schreibersii* was analyzed using microsatellite and mtDNA [59]. It was found an approximately uniform distribution of nuclear DNA (nDNA) alleles in contrast with mtDNA, which showed more geographic structuring [59]. The results indicated a male-biased gene flow and female philopatry at a continental scale. Moreover, it was found a relatively high genetic diversity in western regions such as Iberia and North Africa, which could be explained by an introgression with a recent described species *M. maghrebensis* Puechmaille et al. 2014 from North Africa [59]. No samples from Cabo Verde were included in the published literature, so it is unknown which geographic affinities those individuals, only known from Santo Antão Island, could present.

Little is known about the systematic of the *T. nudiventris*. The distribution is very scattered, it occurs mostly in sub-Saharan Africa along the river Nile, and through the Middle East to southern Turkey, and more arid areas of Indian subcontinent [60,61]. The southernmost distribution report is from northern Tanzania and there are two isolated records from Myanmar. The Middle Eastern part of its range span from Egypt and Sudan through the Levant and Mesopotamia. This species is presently known from Afghanistan, Bangladesh, India, and Pakistan. This species is found in arid and semi-arid zones, tropical forests, and wet evergreen forests [61]. In Cabo Verde, at the western limit of its distribution, it was recorded on Santiago and Maio islands [24]. The genus has been recently studied with molecular markers and two well-supported mtDNA lineages within *T. nudiventris* were confirmed in the Afro-Arabian region highlighting the new of a taxonomic review: one including mostly Iraqi and Iranian samples and another Syrian, Arabian, and African samples [62].

The objective of this paper is to revise the systematic position and distribution of the Cabo Verde bats based on molecular markers, morphological, and acoustic data. More specifically, this paper aims to: (1) Identify the systematic position of Cabo Verde bats in relation to the Macaronesian region, Europe and Africa to test their native or exotic origin; (2) update bat distribution in the Cabo Verde Archipelago based on field work and available museum data to test possible colonization patterns. This study is a breakthrough for starting bat research in that country, while also giving support for conservation actions for bat unique evolutionarily units.

## 2. Materials and Methods 

### 2.1. Study Area

**Figure 1:**
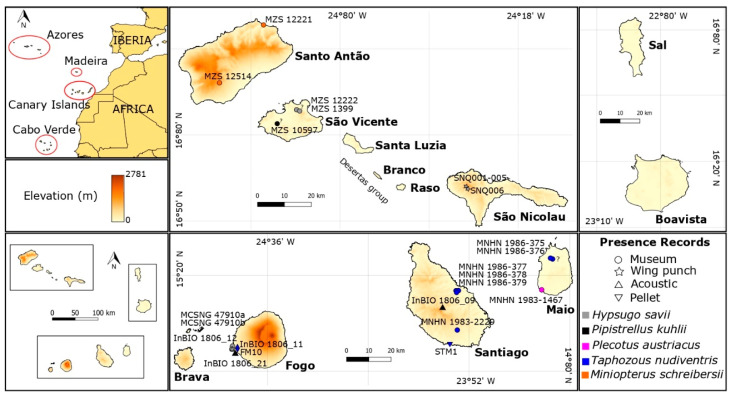
Study area and collected bat presence records. Map of the Cabo Verde Islands, showing the geographic location and elevation of the archipelago and its islands, and bat presence records. Each bats species is depicted with a different color and each sample type with a different shape. Question markers represent unknown locations. See Table S1 for more details.

The Cabo Verde Archipelago is located in the North Atlantic Ocean, close to the West African coast and the West Mediterranean region (Figure 1). The archipelago is formed by ten volcanic islands and several islets situated between 14°45’–17°10’ N and 22°40’–25°20’ W (Figure 1). It lies circa 570 km from African mainland (coast of Senegal), 1500 km from Canary Islands, and 2500 km from Azores, covering a combined area slightly over 4000 km^2^ and spreading over 58.000 km^2^ [63].

The islands are usually classified into three groups: Northern Islands: Santo Antão, São Vicente, Santa Luzia, and São Nicolau; Eastern Islands: Sal and Boavista and Southern Islands: Maio, Santiago, Fogo, and Brava (Figure 1). Moreover, they are also classified as the Windward Islands (Barlavento), which comprised the northwestern and eastern Islands and the Leeward Islands (Sotavento), comprising the southern Islands. Cabo Verde is included in the African Sahelian arid and semi-arid climatic region, experiencing climatic ranges from tropical dry to semi-desertic [63]. Mean annual temperatures range from 23–27 °C at sea level to 18–20 °C at high altitudes, but higher temperatures (35–40 °C) can occur in inner regions of the arid Eastern Islands [63]. Annual precipitations are usually low ranging from 80–300 mm in the arid coastal zones and 1200–1600 mm in the highlands of the mountain islands [63]. Sal, Boavista, and Maio islands have a flat landscape (Figure 1) and a more arid climate because of the infrequent occurrence of rainfall due to their exposure to dry and hot winds coming from the Sahara [63]. All Cabo Verde Islands are volcanic in origin and considered as the result of magmatism generated by ascending mantle plumes under a gradually moving African plate [64]. The volcanic activity probably started sometime in the Late Oligocene or Early Miocene [64]. The age of the islands is not well-known, but most works suggest that islands closer to the mainland are the oldest (12–26 Million years, My), while the most apart are the youngest (2–8 My) [65,66,67]. Brava is thus probably the youngest island, which can be dated to 1.95 ± 0.38 My [68], while Maio is thought to be the oldest with 21.8 ± 5 My [69].

### 2.2. Sample and Data Collection

The fieldwork was performed on São Nicolau Island, at Monte Gordo (16°37’14’’ N, 24°20’36’’ W) in November 2015; Fogo Island, at São Filipe (14°53’42’’ N, 24°29’50’’ W) and Fogo Natural Park (14°59’44’’ N, 24°20’39’’ W) in June 2018; and Santiago Island, at Rocha Preta (15°02’20’’ N, 23°36’18’’ W), Monte Caleirão (15°04’42’’ N, 23°36’26’’ W) and Calabaceira (14°55’52’’ N, 23°35’45’’ W) also in June 2018. All the permits were emitted by “Direcção Nacional do Ambiente” (permit nr. 117/2018). Mist-nets were set up (1 × 9 m, 1 × 10 m, 1 × 6 m) close to the entrance of roost and hibernation sites and pellets were collected for DNA analyses when present. Once a bat was captured, a small piece of the skin was removed for DNA analysis, and all the individuals were released after morphological measurements.

The acoustic sampling was performed every night from 5:30 am to 12 pm with a Pattersson D1000 bat detector with sampling rate of 512 kHz (Pettersson Elektronik AB, Uppsala, Sweden). We set the bat detector during the first period at dusk, which is usually the period of greater activity and until midnight to hear and record echolocation calls and feeding buzz. The sound was recorded using a microphone Zoom H1 Handy Recorder (www.zoom.co.jp) in time-expansion (10 times). In order to identify species, calls were analyzed with the BatSound software (Pettersson Elektronik AB, Uppsala, Sweden). The variables analyzed were frequency of maximum energy (FME), low frequency (LF), high frequency (HF), duration (DUR), and inter-pulse intervals (IPI). The results were compared with the available literature for the five species that occurs in the archipelago. The sounds were deposited at Morphobank (Project number 3514). All coordinates from new observations were recorded and mapped using DivaGiS [70]. 

We also visited the *Museo Civico di Storia Naturale*, *di Genova* (MCSNG), Genoa, Italy, the *Museo Zoologico de La Specola* (MZS), Florence, Italy, and the *Muséum National d’Histoire Naturelle* (MNHN), Paris, France for collecting DNA samples and basic morphological measurements. The vouchers analyzed are represented in Figure 1 and Table S1. Tissue samples were collected using the wing punch method, which involves removing a small circle of the skin (normally smaller than 3 mm) from the wing membrane using a biopsy punch. The morphological measurements were taken using a caliper according to the identification key [71]. The variables used were the lengths of forearm (FA), fifth finger (D5), third finger (D3), thumb (D1), lower leg (Tib), hind foot (HF), length of the 1st and 2nd phalange of the 4th finger (P4.1 and P4.2), and the 2nd and 3rd phalanges of the 3rd finger (P3.2 and P3.3). Both DNA and morphological data collection were performed wherever animals were captured in the fieldwork or the curator allowed it. All measurements were taken following the recommendations proposed by [71]. The photos of the studied specimens are available at Morphobank (P3514).

### 2.3. DNA Extraction, Amplification and Sequencing

The feces were preserved in ethanol 96% and frozen. The DNA from the samples collected with wing punch method was extracted following the standard protocol of DNA extraction with silica. The samples from museums were processed following the ancient DNA extraction protocol and the DNA extraction of wing punches samples was done following the protocol of saline extraction (Supplementary Information Text A).

The mitochondrial marker chosen for this study was the *cyt b*. This gene is widely used in systematic studies to resolve divergences at many taxonomic levels. It has been considered one of the most useful genes for phylogenetic studies and is probably the best-known mitochondrial gene with many sequences on GenBank data base. We designed the *cyt b* primers for each species (see Table S2). The nuclear gene chosen was the *RAG2*, widely used as phylogenetic marker with highly heterogeneous base composition.

The PCR for samples from museums and pellet (PCR I) were carried-out in volumes of 12 μL, comprising 5 μL of PCR Master Mix, 0.4 μL of forward primer, 0.4 μL of reverse primer, 2.2 μL of ultra-pure water, and 4 μL of DNA extraction. Cycling conditions used an initial denaturing at 95 °C during 15 min, followed by 55 cycles of denaturing at 95 °C for 30 s, annealing at a gradient temperature ranging from 46–54 °C during 50 s, and extension at 72 °C for 90 s, and final extension at 72 °C for 10 min. To amplify a fragment of the *RAG2*, we used the primers RAG2-F1 and RAG2-R2 (see Table S2). After the first PCR, a nested PCR was done with the primers RAG2-R1 and RAG2-F1int [72]. The PCR reactions (PCR II) were carried-out in volumes of 10 μL, comprising 5 μL of PCR Master Mix, 0.4 μL of forward primer, 0.4 μL of reverse primer, 2.2 μL of ultra-pure water, and 2 μL of DNA extraction. Cycling conditions used an initial denaturing at 95 °C for 15 min, followed by 35 cycles of denaturing at 95 °C for 30 s, annealing at a gradient temperature ranging from 50–53 °C for 50 s and extension at 72 °C for 90 s, and the final extension at 72 °C for 10 min. For the *cyt b* fragment of *Hypsugo savii*, we used the primers CB1 and cytb2 for the first fragment [73] and cb2F and CB3H for the second fragment [74] (see Table S2). The PCR conditions for samples of *H. savii* for this gene (PCR III) were carried-out in volumes of 10 μL, comprising 5 μL of master mix, 0.4 μL of each primer, 2.2 μL of ultra-pure water, and 2 μL of DNA extraction. Cycling conditions used an initial denaturing at 95 °C during 15 min, followed by 35 cycles of denaturing at 95 °C for 30 s, annealing at 50°C during 50 s and extension at 72 °C for 90 s, and final extension at 72 °C for 10 min.

In order to proceed the Sanger sequencing, 8 μL of the amplified product of the PCRs were transferred to a new PCR plate and 1 μL of EXO + SAP (24 μL of EXO and 96 μL of SAP) was added. The samples were cleaned with the following thermocycling program: 37 °C for 15 min, 85°C for 15 min, and 10 °C for infinite holding. The sequencing reactions were carried-out in volumes of 10 μL, comprising 7 μL of ultrapure water, 1 μL of TRR buffer, 0.5 μL of TRR, 0.5 of primer and 1 μL of the EXOSAP reaction product. The initial temperature was 94 °C during 3 min, followed by 25 cycles of 96 °C for 10 s, 52 °C for 0.05 s, and 60 °C for 4 min. The samples were cleaned with 400 μL of Sephadex per sample, and sequenced amplified from both strands on an automated Sanger sequencer.

### 2.4. Phylogenetic and Population Analyses

After sequencing, every fragment was checked in GenBank using Basic Local Alignment Search Tool (BLAST) to find regions with higher similarity. The sequences were align using ClustalW multiple alignment application [75] and edited in the Geneious Prime v2019.2 (www.geneious.com). Each fragment was inspected visually for ambiguities. Sequences were trimmed for each marker to a fragment in which peaks could unequivocally be assigned for all individuals. Incongruent sequences, stop-codon/ indels, and double peaks were corrected manually to minimize alignment gaps. The uncertainty positions were filled up with the IUPAC nucleotide ambiguity codes. The amplification success varied greatly among samples because of the differences in the quality of the DNA with regard to the conservation conditions and origins. The dataset used for the phylogenetic analyses of the gene *cyt b* consisted of an alignment of 702 base pairs (bp) for 38 *Hypsugo* specimens and four *Pipistrellus* used as outgroup based on published evidence [76]. The phylogenetic analyses of the gene *cyt b* for *T. nudiventris* consisted of an alignment of 278 bp for 42 *Taphozous* specimens*,* and *T. perfomatus* was used as outgroup. For the network of *Miniopterus* we used 114 bp of *cyt b* and 162 sequences with similarity in BLAST higher than 97% (see Table S3 for more details). 

In order to select the best-fit models of evolution to each dataset the JModelTest v2.1.10 was used [77]. The best-fit models of evolution were selected according to Akaike Information Criterion (AIC). Phylogenetic analyses were performed using maximum likelihood (ML) and Bayesian inference (BI) analyses. ML analyses were performed with PhyML v3.0 [78]. A GTR+I+G model was used for *Hypsugo,* and TN93 for *Taphozous*, and reliability was assessed by 1000 bootstrap replications. BI analyses were conducted using BEAST v2.6.1 [79], using the *Hypsugo* dataset. These analyses were used to infer the phylogenetic relationships and simultaneously estimate the timing of the cladogenese events. All parameters were established using the extension BEAUti v2.6.1 [79]. The lack of internal calibration points in *Hypsugo* could not provide a realistic calibration for shallow divergences between sibling species. Therefore, the mean substitution rate of the *cyt b* mitochondrial gene calculated for the genus *Myotis*, family Vespertilionidae was used for this purpose [80]. The molecular clock estimation of nodes ages was chosen because of its similarities with *Hypsugo* and pipistrelles in genetic divergence and thus expected divergence time and taxonomic proximity. The BI was conducted with Yule process as tree prior and random starting trees without constraints, simulations of Markov Carlo Mont Chain (MCMC) were run for 50 million generations, trees were sampled every 1000 generations. Stationarity was assessed by examining the standard deviation of split frequencies and by plotting the -In L per generation using TRACER v1.7 [81] and trees generated before stationarity were discarded as “burn-in.” The models were tested in addition to all options for the clock models (strict, uncorrelated relaxed lognormal, gamma and exponential, random local and fixed local) with likelihoods compared in TRACER v1.7. TreeAnnotator application [79] was used to summarize the information from a sample of trees produced by BEAST onto a single tree with information about posterior probabilities of the nodes and estimates of highest posterior density intervals. Then, the file generated was open in the FigTree v1.4.4 (http://tree.bio.ed.ac.uk/software/figtree) to have a graphical viewer of the phylogenetic tree. 

The genealogical relationships among taxa were assessed with haplotype networks constructed using statistical parsimony, implemented in the program TCS v.1.21 [82] with connection limit 95% and deletions treated as a fifth state. The program tcsBU [83] was used to improve the final network layout. The genetic population analyses were performed in MEGAx v10.0.5 [84] and DNASP v.6 [85] to check the value of number of polymorphic sites, number of haplotypes, haplotype diversity, nucleotide diversity, and within mean group distance of each population.

## 3. Results

### 3.1. Collected Data and Samples

We were able to collect the following samples from museums: MCSNG 47910a, MCSNG 47910b, MZS 1399, MZS 12222 (*H. savii*), MZS 12221, MZS 12514 (*M. schreibersii*), MZS 10597 (*P. kuhlii*), MNHN 1986-375, MNHN 1986-376, MNHN 1986-377, MNHN 1986-378, MNHN 1986-379, MNHN 1983-2229 (*T. nudiventris*), and MNHN 1983-1467 (*P. austriacus*) (Figure 1 and Table S1). We could amplify a small fragment of every sample except the MZS 1399, MNHN 1986-377, and MNHN 1983-1467.

We were unable to amplify DNA fragments of *P. austriacus* held in the Natural History Museum of Paris, which is the only known specimen from Cabo Verde as the DNA was degraded. The only specimen of *P. kuhlii* held on the Museo Zoologico de La Specola, Firenze (MZS) is actually *H. savii.* The specimen was previously identified based only on morphological analyses, and we identified it using molecular and morphological tools.

The samples collected using the wing punches method from fieldwork expedition had great DNA quality and we were able to amplify a larger fragment of the *cyt b* and *RAG2.* The sample SNQ006 was the only one in which we could not amplify for the *RAG2* marker. All sequences were deposited in GenBank with accession codes MT821906–MT821906 (see Table S3 for more details).

### 3.2. Acoustic Analyses and Morphological Data

**Figure 2:**
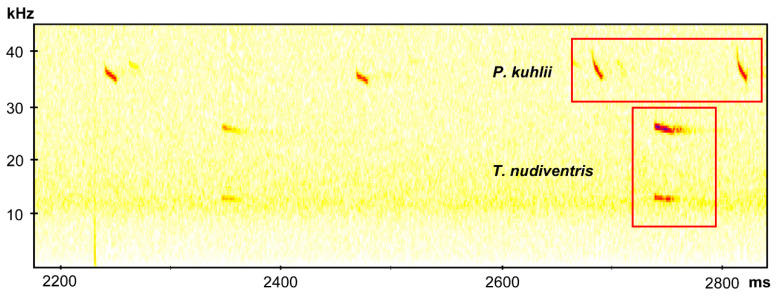
Results of acoustic analyses. Examples of the recorded sonograms for *Pipistrellus kulli* (higher frequency) and *Taphozous nudiventris* (lower frequency). These and other records are available in Morphobank (P3514); see Table S1 for more details.

Echolocation calls were recorded during free-flight and we could identify a typical frequency modulation (FM) and quasi constant frequency (qCF) components of *P. kuhlii* calls for Santiago and Fogo islands. The peak frequency was around 39–42 kHz, the pulse duration was about 6 ms, and the interval between pulses was between 100 and 200 ms (Figure 2). We were also able to identify the species *H. savii* for Fogo Island. This pulse was characterized by a CF with an explosive start, peak frequency around 36 kHz, and pulse duration of 12 ms (Figure 2). The species *T. nudiventris* also appears in the records for Fogo Island, and was characterized by the CF pattern and the peak frequency around 24 kHz, interval between pulses of 400 ms and a pulse duration of 20 ms (Figure 2). The latter represents the first record of *T. nudiventris* species for Fogo. These recording were deposited at Morphobank (P3514). One pellet sample collected on São Filipe was amplified and checked at BLAST, it was correspondent to *T. nudiventris*, corroborated the presence of the species on Fogo Island. The morphological data collected from vouchers and São Nicolau specimens are represented in Table 1.

### 3.3. Phylogenetic and Population Analyses

**Figure 3:**
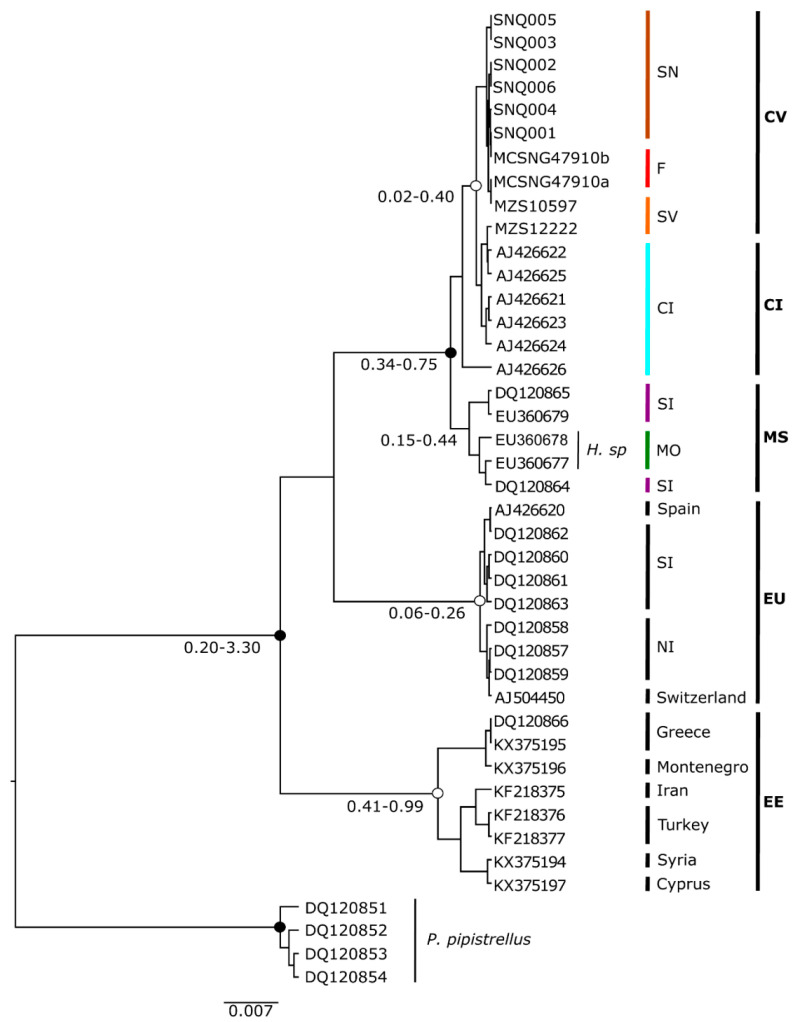
Phylogenetic results for *Hypsugo* samples from Cabo Verde based on the *cyt b* mtDNA sequence variation. Maximum likelihood (ML) phylogenetic tree of *Hypsugo* individuals rooted with *P. pipistrellus* (outgroup). Black dots on the nodes indicate both ML bootstraps values ≥70 and Bayesian inference (BI) posterior probability ≥ 0.95, while white dots show BI ≥ 0.95. The 95% highest posterior density intervals of the nodes estimated with BEAST dating analysis are indicated below the branches. Clades of Cabo Verde (CV) — São Nicolau (SN), Fogo (F) and São Vicente (SV) islands; Canary Islands (CI); Morocco and Southern Iberia (MS) — Morocco (MO) and Southern Iberia (SI); Europe (EU) — SI and Northern Iberia (NI), and Eastern Europe (EE) — are colored according to their geographic location corresponding to Figure 4. For further details on the samples, see Figure 1, and Table S1 and S3.

The phylogenetic tree constructed for estimating the relationships of *Hypsugo* from Cabo Verde with related groups is presented in Figure 2. The phylogeny with highest likelihood was obtained from Yule species model and strict molecular clock (likelihood= −2255). The results of ML and BI analyses yielded the same topology, with high bootstrap values and posterior probabilities in most clades (Figure 3). The Cabo Verde samples presented some geographical structure. The sample MZS 12222 from São Vicente was the only sample from Cabo Verde that nested within the Canary Islands clade. The Cabo Verde and Canarian clades split around 0.049 million year ago (Ma). The Cabo Verde samples grouped together with *Hypsugo* from Canary Islands in a monophyletic well-supported group (BI posterior probabilities >95%; Figure 3). The sample AJ426626 from El Hierro Island represented a slightly different lineage [33]. Nevertheless, it also nested in the Canarian clade with a BI posterior probabilities superior to 90%.

The samples of *Hypsugo sp*. from Morocco and two samples of *H*. *savii* from Southern Iberia formed a well-supported sister clade of *Hypsugo* from Cabo Verde and Canary Islands (Figure 2). However, this clade is polyphyletic. The group of Cabo Verde, Canary Islands and the samples from Southern Iberia and Morocco split about 0.65 Ma. The other group is represented by samples from mainland Europe (Iberian Peninsula and Switzerland), with two clear subgroups with geographic structure: one of samples from southern Iberia, and another with samples from northern Iberia and Switzerland that split circa 0.22 Ma (Figure 3). Finally, the Eastern Europe clade composed by sequences from Greece, Montenegro, Iran, Turkey, Syria, and Cyprus also present a clear geographic grouping of samples and split from the other clades around 0.7 Ma (Figure 3). 

**Figure 4:**
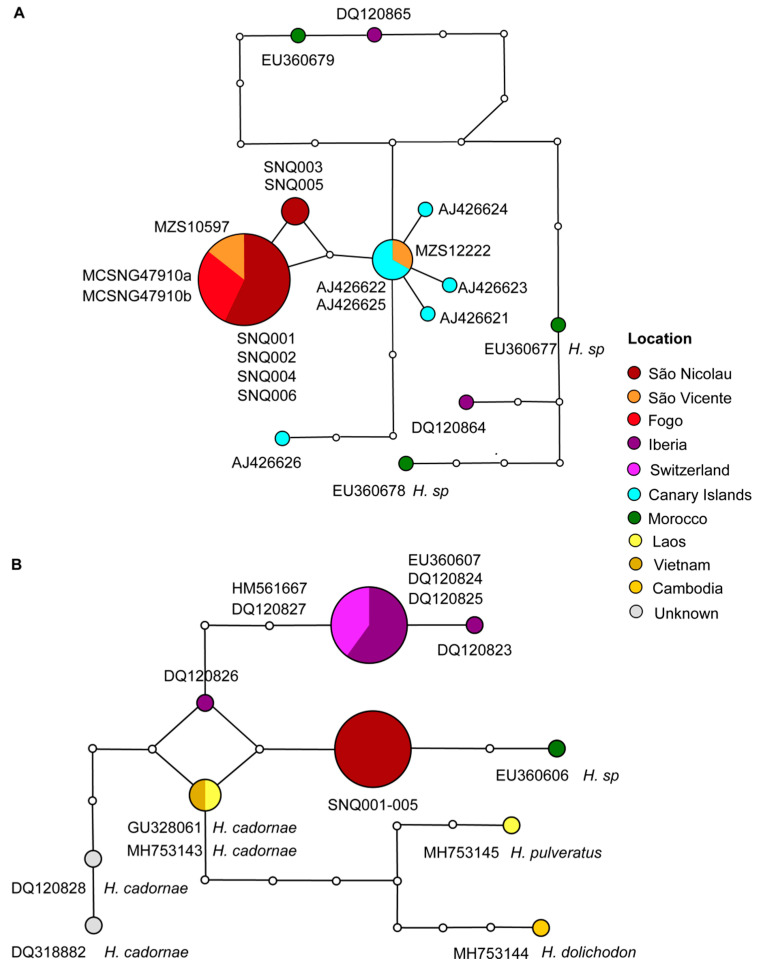
Parsimony networks including sequences similar to those of *Hypsugo* from Cabo Verde Islands. Network corresponding to the *cyt b* mtDNA (**A**) and *RAG2* nDNA sequences variation (**B**). Lines represent mutational steps, circles indicate haplotypes, and dots show missing haplotypes. The size of the circle is proportional to the number of individuals, and different colors correspond to different locations. For further details on the samples, see Figure 1, Table S1 and S3.

Regarding the *cyt b* haplotype network of *Hypsugo* (Figure 4), the results showed one haplogroup corresponding to Cabo Verde samples, which is composed of two haplotypes unique from Cabo Verde. One of them is more common, being present in Fogo, São Vicente and São Nicolau islands, and the other composed by samples only from São Nicolau Island (Figure 4). However, there is a third haplotype present in Cabo Verde samples. The latter one is closely related to the Canarian haplogroup, which showed five haplotypes. The sample MZS 12222 from São Vicente exhibit the same haplotype of the most frequent haplotypes from Canary Islands (Figure 4). Considerable differentiation was found in the sequence from El Hierro Island (AJ426626), which exhibit four mutational steps, being more distant than the haplogroup of Cabo Verde (two mutational steps). The samples from mainland Europe and Africa showed differentiation by five mutational steps from both haplogroups. The haplotype network of *RAG2* corroborates with the view that sequences from Cabo Verde correspond to a unique haplotype, two mutational steps apart from *H. sp.* from Morocco, *H. savii* from Switzerland, and *H. cadornae* from Laos and Cambodia (Figure 4).

A total of 37 polymorphic sites and 23 haplotypes were found in *Hypsugo* sequences (Table 2). The genetic diversity of each population regarding the haplotype (Hd) and nucleotide diversity (π) was high in the population of Canary Islands (CI) and the group of Morocco and South Iberia (MS) samples. The genetic diversity was relatively high in Europe (EU) and Eastern Europe (EE) populations (Table 2). The haplotype diversity of the Cabo Verde (CV) population was 0.200 (± 0.154) and the nucleotide diversity 0.00063 (± 0.0048). The value of evolutionary divergence between groups showed that the most divergent was the EU clade when compared with MS. The closest related population was the CV and CI (Table 2).

**Figure 5:**
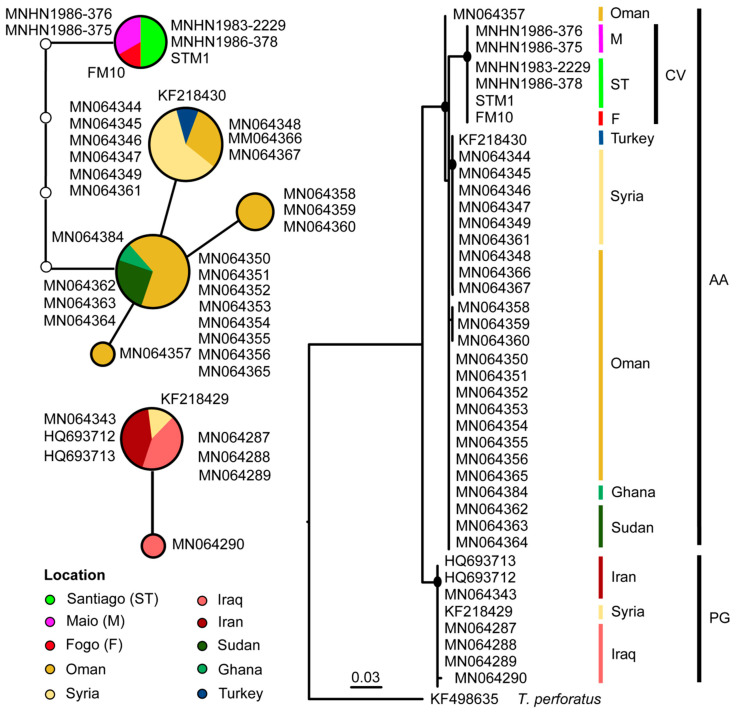
Parsimony network corresponding to the *cyt b* mtDNA sequences in *Taphozous nudiventris*. Lines represent mutational steps, circles indicate haplotypes, and dots show missing haplotypes. The size of the circle is proportional to the number of individuals, and different colors correspond to different locations. The evolutionary history was inferred by using the maximum likelihood (ML) phylogenetic tree rooted with *T. perforatus* (outgroup). Black dots on the nodes indicate ML bootstraps values ≥ 70. Clades (M, Maio; ST, Santiago; F, Fogo; CV, Cabo Verde; AA, Afro-Arabian clade; PG, Persian Gulf clade) are colored according to their geographic location. For further details on the samples, see Figure 1, Tables S1 and S3.

For *T. nudiventris cyt b* alignment, the results of ML and network analyses are represented in Figure 5. Samples from Cabo Verde formed a monophyletic well-supported clade (bootstrap values > 70) closely related to the Afro-Arabian lineage with samples from Ghana, Sudan, and some of Oman (Figure 5). The *cyt b* network showed that Cabo Verde samples from three different islands have the same haplotype separated by five mutational steps from the African and Arabian samples (Figure 5). It was also evident that the individuals from near the Persian Gulf, namely from Iraq, Iran, and one sample from Syria, form a different network with differences above the selected 95% connection limit (Figure 5).

**Figure 6:**
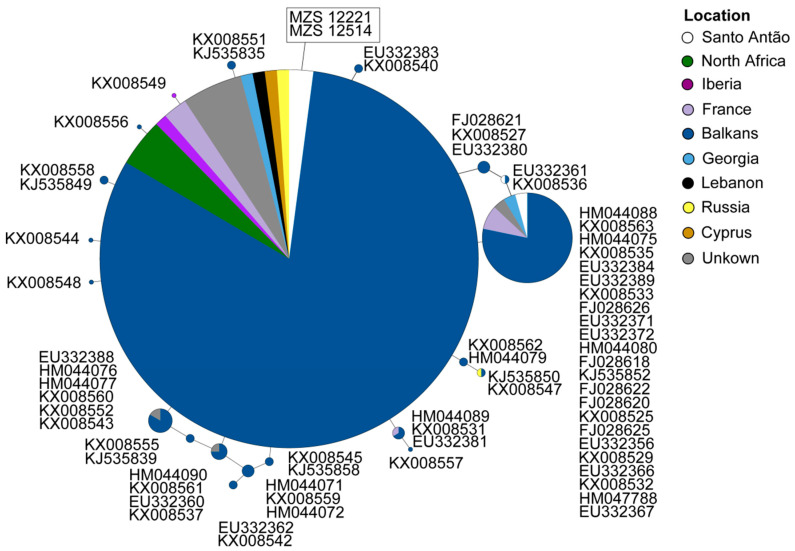
Parsimony network corresponding to the *cyt b* mtDNA sequences in *Miniopterus schreibersii*. Lines represent mutational steps, circles indicate haplotypes, and dots show missing haplotypes. The size of the circle is proportional to the number of individuals, and different colors correspond to different locations. The only samples from Cabo Verde are within a rectangle and correspond to the main central haplotype. For further details on the samples, see Figure 1, Table S1 and S3.

Regarding the *cyt b* network of *Miniopterus*, 21 haplotypes were found. The Santo Antão sample shared the same and most common ancestral haplotype with samples from Russia, Cyprus, Lebanon, France, Iberia, North Africa, and Balkans (Figure 6).

## 4. Discussion

This study presents the first phylogenetic and network reconstruction of the *Hypsugo*, *Taphozous,* and *Miniopterus* for the Cabo Verde Archipelago. It also expands the geographic distribution of *T*. *nudiventris* to Fogo Island and represent new morphological and acoustic data for this and the other bats species that may be useful for future systematic studies. 

Our results based on mtDNA and nDNA corroborate the presence of a divergent lineage of *H*. *savii* complex in the Western Palearctic detected in previous studies [12], which are spread across Canary Islands and North Africa to Turkey [36]. Samples of *Hypsugo* from Cabo Verde Islands do not share haplotypes with specimens from Canary Islands but form a monophyletic clade with those (see Figure 2 and Figure 3). These results point to an independent colonization event of Cabo Verde by Canarian ancestral about 0.049 Ma. However, one specimen from São Vicente Island (MZS 12222) grouped with individuals from Gran Canaria (AJ426622) and La Gomera (AJ426625). This specimen could have reached Cabo Verde by its own means, suggesting a second recent colonization event or it could have been introduced in the archipelago by passive transport from the Canary Islands, thus explaining no genetic differentiation between them. The migratory behavior of *H*. *savii* is unknown [86]. The species may be good at colonizing islands, as it occurs in many Mediterranean islands, and it also has been reported as a vagrant in the Great Britain [87]. In the Cabo Verde Archipelago *Hypsugo* was reported for northwestern and southern islands [24]. In this study, we prove that individuals from both island groups share the same haplotype, supporting the hypothesis of gene flow among relatively long-distanced populations. Thus, *Hypsugo* individuals from Southern Iberia Peninsula may have colonized the North of Africa by crossing the geographic barrier of Gibraltar Strait, and then the Canary and Cabo Verde Islands by stepping-stone in the Saharan Islands, existing between both archipelagos during the Pleistocene glaciations [88]. In fact, two samples of *H*. *savii* from Southern Iberia were found clustering within the North African lineage [13]. For this species, the Strait of Gibraltar represents a barrier in the distribution of lineages because it showed distinct haplotypes on each side of the Strait. However, some permeability occurs in a very low proportion of the haplotypes from Iberia that clustered within the Moroccan lineages [13]. Instead, *Hypsugo* individuals from Italian peninsula could have reached the north of Africa using Sicily as stepping-stone, as suggested by *ND1* mtDNA data [10] and then the Canary and Cabo Verde islands. As, unfortunately, we could not include Italian specimens in our mtDNA analyses, neither Canarian nor Italian specimens in our nDNA analyses, it was impossible to choose one between the two alternative scenarios. Alternatively, they could have reached the archipelago by passive transport, spread by boat traffic, because of their high affinity to roost and forage in synanthropic conditions [87]. The latter scenario is harder to accept because of the old age of the split between the Canarian and Cabo Verdean clades. However, those conditions could have facilitated a second wave of colonization, as reflected in the haplotype sharing with the Canary Islands and facilitated the movement among northern and southern islands, explaining the reduced geographical structure.

The genetic divergence between Cabo Verde samples and the Canary Islands, even though low compared to other major clades, suggested that those lineages should be treated, at least, as different ESUs. A significant differentiation between the Canarian population compared with mainland Spain, in particular on El Hierro Island, was previously found [33] which showed more distant haplotypes within the Canarian haplogroup than from the most common Canarian haplotype to samples from Cabo Verde. Therefore, either there are more than one undescribed taxa on Canary Islands, or Canarian and Cabo Verdean populations should be described within the same taxon. As explained in detail in the introduction, some authors recognized *H. savii* as two or even more species [12,45,89]. There is a current discussion regarding the taxonomic status of *Hypsugo cf*. *darwinii*, which was first described to the North-Africa/ Canarian group. In 2007, provisional use of the name *Hypsugo cf*. *darwinii* for the Canary Islands and Morocco *Hypsugo* individuals based on significant mtDNA differences with respect to European mainland individuals was suggested [12]. Later, *H*. *darwinii* was treated as a separated species [89] and stated that it “resembles” most closely the Savi’s pipistrelle bat of Northern Africa, the Canary Islands, Sicily, and Sardinia [36]. In the absence of formal designation of *H*. *darwinii* as a separated taxon, the species is currently not accepted as occurring in Europe. The fact that most Cabo Verde specimens presents unique haplotypes opens the question of considering them as a different taxon, as they are as distant to the mainland European *Hypsugo* than individuals from Canary Islands, or treat them within *H*. *darwinii*. Our mtDNA and nDNA results support the validity of a Macaronesian-Magrebian taxon as a solution to partially resolve the current *H*. *savii* species complex. 

Unfortunately, other lines of evidence do not help clarify the taxonomic status of *Hypsugo* individuals from Cabo Verde. The echolocation records of *Hypsugo cf*. *darwinii* are closely related to those of *H. savii* [37,90,91,92]. These two taxa seem to echolocate using almost identical calls, increasing the difficulties to identify *H*. *cf*. *darwinii* based on only acoustic surveys. Moreover, the identification through morphological measurements is difficult because of the lack of a modern morphological description [37]. Hair coloration is usually used as a taxonomic character, and *H*. *cf*. *darwinii* apparently has a dorsal lighter brown pelage with a small reddish-brown spot between the ears, the corners of mouth, and the shoulders [37]. The specimens caught in São Nicolau have a dark and long dorsal pelage with contrasting light golden tips. However, the reddish-brown spot is not as evident as reported by other authors [37] (see photos on Morphobank). Another character used to distinguish *Hypsugo* is the size and shape of the ear and tragus. Tragus are short and slightly broadening above, the length of the front margin of the tragus almost corresponding to its width, and the tip of the ear is broadly rounded in *H*. *savii* [71]. Unfortunately, we could not compare the morphological measures of the museum specimens to the individuals caught in the field. The preserved material was not in a good condition. The length of the forearm, fifth and third finger is within the range expected for *H*. *savii* from Iberia. Other measurements are not discriminative between *H*. *savii* and *H*. *cf*. *darwinii*.

The global wind circulation pattern may explain the routes of colonization for flying animals. The pattern of the colonization of Cabo Verde Archipelago may have occurred from north to southwestern from the Canary Islands. Thus, the most likely hypothesis is that the northern islands as Santo Antão were the first to be colonized followed by stepping-stone colonization to other northwestern islands. In fact, there is an unconfirmed record of *Hypsugo* on Santo Antão [24] already present in all other northwestern Cabo Verde Islands, except the extremely dry Desertas Islands. Although Sal and Boavista islands are closer to the African mainland, few observations of bat were recorded, and all probably of vagrant individuals and none *Hypsugo* specimen [24]. In fact, the migratory *E*. *helvum* is a common species broadly distributed across the lowland rainforest and savanna zones of Africa from Senegal in the west, through to South Africa in the south and Ethiopia in the east. However, only a single individual was found in Cabo Verde, Boavista [32]. These islands appear to have an unsuitable environment for the referenced bat species, as the climate is drier, and the terrain is flatter compared with the northwestern and southern islands (Figure 1). Little is known about the habitat requirements for *H*. *savii/ H*. *darwinii*, but these are found mainly in uplands and mountains, forages over open woodland, pasture and wetlands, which may explain the absence of *Hypsugo* records on those eastern islands.

Regarding the phylogeny of *T. nudiventris*, it seems clear that Cabo Verde population is further apart from the Persian Gulf lineage than to the Afro-Arabian lineage previously detected [62], but the lack of samples from Western mainland Africa precludes any further biogeographical interpretation of our results. The fact that Cabo Verde individuals present all the same unique haplotype could be interpreted as recent colonization of the islands or introduction of individuals from any of those closely related Afro-Arabian geographical areas. It could be also the result of unsampled intra-archipelago diversity. When analyzing the distribution pattern of *T. nudiventris*, it appears to occur only in the southern islands as there is no record for this species in the northwestern or eastern islands until now. However, the distribution range of this species in the archipelago could be greatly underestimated because of the poor number of bat surveys in the country. In fact, in our acoustic data we found a typical call of *T*. *nudiventris* on Fogo Island. This record, together with pellet samples, expanded the geographic range for this species westward. As this species is a typical representative of the African mainland, the most possible scenario is that the first islands to be colonized were the eastern or northwestern islands. This species is found in arid and semi-arid regions, and populations in northern Africa are found in Sahelian and Sudanian savanna zones [61]. The close-relatedness with individuals from Ghana and Sudan support this scenario. The desert-like characteristics of Sal and Boavista and other dryer northwestern islands, such as São Vicente and the Desertas might provide a suitable habitat for *T*. *nudiventris* and settlement for immigrants from the mainland that could present different haplotypes from the ones sampled if speciation occurred. In previous studies that analyzed the genetic structure of other flying vertebrates (kestrel), a reduced gene flow between Leeward and Windward populations was found [93], and the same pattern was found in reptiles [94]. Because of the lack of samples from Leeward Islands, we are hence unable to distinguish if *Taphozous* population of Cabo Verde represents an exotic native or taxa, which would represent diametrically opposite results for conservation purposes.

The shallow genetic differentiation in *M. schreibersii* previously found [95] and posteriorly confirmed [59,96] by other authors suggests a recent and rapid postglacial re-colonization of Europe, probably from Anatolia acting as a single glacial refugium, which showed the highest allelic richness [59,96]. Unlike the pattern of *H*. *savii*, *P*. *kuhlii,* and *P*. *auritus*, which re-colonized Europe from two glacial refugia, the extant population of *M. schreibersii* seems to be originated from a single location. The lack of structuring among populations of *M. schreibersii* in a continental scale indicates that there is no isolation-by-distance, which is unexpected for a small mammal like a bat on a large geographic distribution. It is known that *M. schreibersii* is a volant migratory species, with wings adapted for long-distance flight in high-altitude [97]. The Gibraltar Strait appears to have no effect on dispersal rate for this species, which shows no genetic discontinuities between Europe and North Africa [13]. However, the species also exhibits philopatric behavior. The mtDNA showed more geographic structuring than the autosomal microsatellite markers, suggesting a male-biased dispersal and female philopatry [59,96]. Our results showed that *Miniopterus* from Cabo Verde has the same haplotype as *M. schreibersii* from Western and Eastern Europe, Middle East, Africa, and Asia. This could, in fact, represent a shallow differentiation between all populations in a continental scale, or even a recent expansion postglacial maximum. 

Regarding the presence *P. kuhlii* in Cabo Verde, we have demonstrated that the sequence of the voucher held in the *Museo Zoologico de La Specola*, Florence, Italy (MZS 10597) matched *H. savii* species. The misidentification is understandable as both species are difficult to distinguish even with fresh biological samples. The skull was not available for teeth analysis, and most diagnostic characteristics used to discriminate them, such as ears, hair, and dorsal pelage were ruined. Thus, the only evidence for the presence of *P. kuhlii* in Cabo Verde now is the records of echolocation calls, which could be easily misclassified with *P. hesperidus* or some Neoromicia bats. Alternatively, the sequenced sample could be contaminated with *H. savii.* Hence, more sampling and collection is needed to confirm the occurrence of *P. kuhlii* in the archipelago. Regarding *Plecotus* bats, there are no clear morphological discriminatory characters described for Cabo Verde population, although the measurements of forearm and thumb of a specimen held at the National Museum of Natural History in Paris confirmed it does not belong to *P. t. teneriffae* or to the Afro-Mediterranean population [54].

Additional sampling in Cabo Verde is essential to study the levels of genetic diversity in the archipelago and between islands. For *Hypsugo*, samples are required from the northern islands, mainly on Santo Antão where there are no confirmed records. However, is likely that the species occurs in this island because of the habitat similarities and proximity to São Vicente and São Nicolau. In the southern islands, more capture effort is necessary on Santiago Island. *Hypsugo* has similar environment requirements as *Pipistrellus*, so the latter species might also occur in this island. This would expand the geographic range for this species in the archipelago and may clarify the genetic diversity within the archipelago. Additional sampling is also necessary on Canary Islands, especially regarding nDNA. Our results based on mtDNA showed that Cabo Verde samples have a unique haplogroup, but one sample is included in the Canary Islands haplogroup. However, our results of nDNA lack information of this archipelago. In order to have a better picture of the genetic structure of *Hypsugo* species complex, more samples from Morocco, Iberia (both northern and southern), Italy (including the Mediterranean islands of Sicily and Sardinia), and the Balkans, mainly from Turkey, are required. Our results revealed that *Hypsugo* from Cabo Verde is closely related to *Hypsugo* from Canary Islands, strongly suggesting that they represent different species. For future studies, the taxonomic status of *Hypsugo cf*. *darwinii* should be clarified in order to proceed conservation and ecological studies. Regarding *Taphozous,* additional information on the African populations, especially from the western mainland, is crucial to identifying the potential origin of the distinct lineages in Cabo Verde. In the archipelago, there are records only on the southern islands. However, the species might also occur in the eastern islands. Thus, more fieldwork, mainly on Sal and Boavista is fundamental to establishing the geographic range and better understanding the genetic structure within the archipelago. Regarding *Miniopterus*, unfortunately the DNA fragment amplified was very short to distinguish different haplotypes, because of the limitations of using voucher species. Because of that, we are not able to properly establish the genetic relationships related to the lineages from western and Eastern Europe, Middle East, Africa, and Asia. Fresh samples would allow higher phylogenetic resolution. As *M. schreibersii* exhibits a philopatric behavior that is crucial to analyze mtDNA and nuDNA with different makers to investigate its genetic structure and diversity.

## 5. Conclusions

Recognizing distinct lineages is fundamental for the conservation actions and little is known about the phylogenetic relationships and the genetic diversity of species present in Cabo Verde. The archipelago may host unique evolutionary lineages that are not yet recognized. In this study, we emphasize on the importance of phylogenetic studies of bats in the Cabo Verde Islands, because they may be the only native mammals in the archipelago. We also highlight the importance of systematic status establishment as the first step for species conservation. This article presents the first phylogenetic reconstruction of Cabo Verdean bats, and also expands the geographic distribution of *T. nudiventris* inside the archipelago. It also discusses the systematic status of *Hypsugo* from Macaronesia and North Africa, highlighting the importance of systematic as the first step to establishing a good conversation plan. 

The scattered distribution of bats in Cabo Verde could be explained by recent colonization or introduction events. Most species did not have enough time to differentiate and colonize other islands. Individual might have reached the archipelago involuntarily by boats or storms [26,98]. The bat colonization could be, in fact, only a few centuries old, considering the recent human occupation of the islands in the 15th, 16th, and 17th century. Alternatively, it is possible that adaptation to an arid oceanic island could have led to speciation of native cryptic species because of the large distance from the Africa mainland and between islands [53]. Poor sampling effort also could explain their scattered distribution. In fact, the bat fauna has been continuously neglected in biodiversity surveys because of the difficulty to capture and identify bat species due to their low abundances and reduced habitat availability. Each new sampling in the archipelago results in new occurrence records for some species or even new observations of Chiroptera taxa for the archipelago (e.g., [24]). Future studies are very important to fill the sampling gaps and to understand better the systematic position of chiropteran species.

## Figures and Tables

**Table 1:** Morphological measurements of specimens. Information about species (Sp), sex (S), length of forearm (FA), fifth finger (D5), third finger (D3), thumb (D1), lower leg (Tib), hind foot (HF), 1st and 2nd phalange of the 4th finger (P4.1 and P4.2), and the 2nd and 3rd phalanges of the 3rd finger (P3.2 and P3.3) are also given. *Hs* stands for *H. savii*, *Ms* for *M. schreibersii*, *Pa* for *P. austriacus*, and *Tn* for *T. nudiventris*.

Sp	Code	S	FA	D5	D3	D1	Tib	HF	P4.1	P4.2	P3.2	P3.3
*Hs*	MZS 1399	F	37.6	32.2	35.5	8.8	16.6	6.6	10.0	8.0	-	11.1
*Hs*	MZS 12222	F	35.4	33.2	35.5	8.8	15.4	6.6	11.1	8.8	-	10.0
*Hs*	MZS 10597	F	35.4	32.2	33.3	8.8	14.4	6.6	11.1	7.7	-	10.0
*Hs*	MCSNG 47910a	M	35.8	31.5	34.2	7.0	15.7	5.0	10.6	10.4	-	11.9
*Hs*	MCSNG 47910b	F	34.5	29.4	30.7	7.5	14.9	5.6	10.7	8.0	-	10.0
*Hs*	SNQ001	M	34.9	43.6	58.0	3.1	15.2	4.6	10.5	10.5	11.0	6.0
*Hs*	SNQ002	F	37.1	52.8	61.3	4.2	16.0	4.8	10.5	10.5	11.0	7.0
*Hs*	SNQ003	M	35.4	42.2	58.3	3.1	15.7	4.7	10.3	10.3	11.2	6.6
*Hs*	SNQ004	M	35.3	43.1	58.4	4.6	15.5	4.5	10.6	9.7	10.7	5.2
*Hs*	SNQ005	M	35.3	43.4	60.2	3.7	15.2	4.0	11.0	10.3	11.0	6.8
*Hs*	SNQ006	M	35.5	32.2	34.4	4.2	15.4	4.3	10.5	8.9	10.1	4.8
*Ms*	MZS 12221	F	43.3	36.6	41.1	9.9	19.9	9.9	9.9	14.3	-	28.8
*Ms*	MZS 12514	M	45.4	38.7	43.2	9.9	20.0	9.9	9.8	15.5	-	29.9
*Pa*	MNHN 1983-1467	unk	40.3	35.3	37.0	10.0	18.4	9.0	10.1	10.7	11.8	8.0
*Tn*	MNHN 1986-375	unk	68.2	42.7	60.0	17.1	28.2	17.7	14.3	8.3	23.4	-
*Tn*	MNHN 1986-376	F	67.4	43.9	61.9	18.7	27.9	15.9	13.5	8.0	25.0	-
*Tn*	MNHN 1986-377	F	69.9	45.3	64.4	17.8	30.9	16.3	15.1	8.6	32.5	-
*Tn*	MNHN 1986-378	M	69.4	43.1	61.5	17.8	27.3	16.7	13.8	8.4	25.5	-
*Tn*	MNHN 1986-379	F	71.3	47.0	68.3	17.3	30.1	16.7	14.1	7.6	27.2	-
*Tn*	MNHN 1983-2229	M	74.9	48.9	69.5	17.5	31.0	14.2	16.7	9.6	29.0	-

**Table 2:** Genetic diversity parameters. Values calculated for *H. savii cyt b* mtDNA sequences for each clade represented in the phylogenetic tree: Cabo Verde (CV), Canary Islands (CI), Morocco and Southern Iberia (MS), Europe (EU), and Eastern Europe (EE). The number of sequences (N), polymorphic sites (S), and haplotypes (h), as well as haplotype (Hd) and nucleotide (π) diversity, and within mean group distance (WMGD) with respective standard error (SD) are given. The estimates of evolutionary divergence over sequence pairs between clades are also given. Standard error estimates are shown above the diagonal.

Clade	N	S	h	Hd ± SD	π ± SD	WMGD ± SD	CV	CI	MS	EU	WE
CV	10	2	2	0.200 ± 0.154	0.00063 ± 0.00048	0.00 ± 0.00	-	0.0025	0.0043	0.0098	0.0090
CI	6	11	6	1.000 ± 0.096	0.00612 ± 0.00197	0.01 ± 0.00	0.0065	-	0.0033	0.0086	0.0079
MS	5	13	5	1.000 ± 0.126	0.00922 ± 0.00161	0.01 ± 0.00	0.0188	0.0125	-	0.0099	0.0096
EU	9	4	5	0.722 ± 0.159	0.00176 ± 0.00049	0.00 ± 0.00	0.0664	0.0503	0.0711	-	0.0090
EE	8	7	5	0.857 ± 0.108	0.00491 ± 0.00072	0.01 ± 0.00	0.0616	0.0470	0.0668	0.0631	-
CV	10	2	2	0.200 ± 0.154	0.00063 ± 0.00048	0.00 ± 0.00	-	0.0025	0.0043	0.0098	0.0090

## Supplementary Materials

The following are available online at https://www.mdpi.com/2073-4425/11/8/877/s1. Text A: DNA extraction protocols. Table S1: Summary of all Cabo Verde samples used in this study. Table S2: Summary of all primers used in this study. Table S3: Summary of all GenBank sequences used in this study.

## Resumo

O Arquipélago de Cabo Verde apresenta uma das maiores lacunas de conhecimento na distribuição e taxonomia de morcegos do mundo. Trabalhos antigos indicaram que existem cinco espécies classificadas como taxa europeus. Aplicamos a taxonomia integrativa para rever a posição e distribuição sistemática dos morcegos cabo-verdianos com dados moleculares, morfológicos e ecológicos, testar a origem nativa ou exótica destes e inferir possíveis padrões de colonização com base em trabalho de campo e amostras de museus. Os resultados mostraram que o *Hypsugo* de Cabo Verde é muito aparentado ao das ilhas Canárias, cujo estato taxonómico está sob debate, apresentando haplótipos mitocondriais e nucleares únicos. Também ampliamos a distribuição do *Taphozous nudiventris* de para a ilha do Fogo por meio de amostras fecais e identificação acústica, demostramos a existeência de haplótipos únicos para essa espécie e que *Miniopterus schreibersii* partilhava um haplótipo com espécimes europeus, norte-africanos e asiáticos ocidentais. A identificação morfológica e acústica de espécimes cabo-verdianos foi um desafio devido à falta de descrições morfológicas modernas e à semelhança das ecolocalizações dentro do mesmo género. Definitivamente, são necessários mais estudos para determinar a sistemática das espécies de morcegos no arquipélago, mas este trabalho é o primeiro passo para o estabelecimento de acções de conservação dos provavelmente únicos mamíferos cabo-verdianos nativos.
