# Supplementary material for: Bats Out of Africa: Disentangling the Systematic Position and Biogeography of Bats in Cabo Verde
Source: Genes (Basel). 2020 Aug 1;11(8):877. doi: 10.3390/genes11080877 (PMC7464910; doi:10.3390/genes11080877)
Supplement: Supplementary file 1 [file genes-11-00877-s001.pdf]

Supplementary Information

A. DNA extraction protocols. Details for the saline, ancient DNA and silica extractions are given.

Saline extraction protocol

A small amount of tissue was taken and cut into fine pieces using a sterile scalpel blade, the contents were transferred into a labeled 1.5 ml eppendorf tube. Then, 600 μL of lysis buffer and 10 μL of proteinase K were added to each tube. The samples were vortexed several times and incubated at 56°C overnight. After that, samples were put in the fridge at 4°C for 10 min, and 300 μL of ammonium acetate was added. The samples were centrifuged for 15 min at 14000 rpm and kept at 4°C. The supernatant was transferred to a new 1.5 ml eppendorf tube, and 600 μL of ice-cold isopropanol was added and mixed by inverting the tubers several times. The samples were put in the freezer overnight and then centrifuged for 30 min at 14000 rpm. The supernatant was discarded and 1000 μL of ice-cold ethanol (70%) was added and mixed by tapping with the finger at the bottom until the DNA pellet was released. The tubes were centrifuged for 15 min at 14000 rpm. The supernatant was rapidly discarded and the tubes were incubated for evaporation at room temperature until complete evaporation. After that, 50 μL of ultra-pure water was added and the samples were leaved to hydrate at ambient temperature overnight.

Ancient DNA extraction protocol

The samples were cut in small pieces and about 50 mg was added to a 2.0 ml eppendorf tube together with 1 ml of extraction buffer (745 μL H_2O; 9 ml EDTA 0.5 M (pH=8.0); 250 μL Proteinase K 10 mg/ml; 5 μL Tween 20) they were left overnight at 37°C in the incubator. In the next day, the samples were spun for 2 min at maximum speed and the supernatant was transferred to a 50 ml falcon tube containing 10 ml Binding Buffer (23.88 g Guanidine hydrochloride; 30 ml H_2O; 50 ml Isopropanol; 25 μL Tween 20) and 400 μL 3M sodium acetate. A MinElute spin column was separated for each sample and an extension reservoir of a V-spin column was forced into the opening of the MinElute tube. The extension reservoir/MinElute assembly was removed from the collection tube and placed into a 50 ml falcon tube.

The solution with Binding buffer mixture was transferred into the extension reservoir, then it was centrifuged for 4 min at 1500 rpm and then for 2 min at 1500 rpm. The extension reservoir/spin column assembly was placed back into the collection tube and a dry spin was performed for 1 min at 6000 rpm in a centrifuge, then the flow-through was discarded. After that, 750 μL PE buffer was added and the samples were centrifuged at 6000 rpm for 30 s and the flow-through was discarded, this last step was repeated. After that, the spin column was turned and a dry spin was performed for 1 min at 13000 rpm, the spin column was then transferred into a 1.5 ml tube with the cap ripped off. Next, 25 μL TET buffer (~49.4 ml H_2O; 100 μL EDTA 0.5 M (pH=8.0); 500 μL Tris-HCl 1 M (pH=8.0); 25 μL Tween 20) was added on top of the silica membrane and left standing for 5 min before centrifugation at 13000 rpm during 30 s. After that, more 25 μL of TET was added and the centrifugation was repeated. The 50 μL of the extraction was transferred to a fresh tube labeled with sample code.

DNA extraction with silica protocol

The feces were preserved in ethanol 96% and frozen. In order to proceed the DNA extraction, the samples were left in the greenhouse overnight to evaporate de ethanol. The entire process was done with filter tips and a negative control was prepared for the set of samples. The first step was adding 20 ml of PBS solution (6.08 g KH_2PO_4; 8.62 g NaCl and 1000 ml H_2O) in a 50 ml falcon with the scat (entire pellet). The material was wash vigorously with a Pasteur pipette to promote the epithelial cells elution. About 15 ml of the supernatant was transferred to a 15 ml falcon and centrifuged at 4000 rpm during 20 min. After that, the supernatant was discarded and 2 ml of buffer L6 (147.8 g Guanidine Thiocyanate; 25 ml Tris-HCl 1 M (pH=6.4); 10 ml EDTA 0.1 M (pH=8.0); 3.25

ml Tríton X-100 and 250 ml H_2O) was added to the pellet and the solution was vortexed. Then, the samples were incubated overnight at room temperature on constant agitation.

In the next day, they were centrifuged at 4000 rpm at 15 min, the supernatant was transferred to a new 15 ml falcon, 250 μL of silica solution was added and vortexed during 2 min to promote the bond of DNA molecules with silica solution. Then, the solution was centrifuged during 2 min at 4000 rpm. After that, the supernatant was discarded and 2.5 ml of buffer L2 (350 g Guanidine Thiocyanate; 59.2 ml Tris-HCl (pH=6.4); 23.7 ml EDTA (pH=8.0) and 592 ml H_2O) was added to each sample and vortexed until the pellet dissolution. The tubes were centrifuged at 4000 rpm during 2 min, this last step was then repeated in order to clean the DNA solution. Then, 4 ml of ethanol 80% was added to dissolve the entire pellet and the samples were centrifuged again at 4000 rpm during 2 min.

The tubes were left in the greenhouse at 60°C to dry the pellet and remove all the ethanol. The DNA elution was made with 500 μL of ultra-pure water, the material was vortexed to dissolve the pellet and incubated at room temperature. After that, the solution was centrifuged at 4000 rpm during 10 min, the supernatant was recovered and transferred to a new eppendorf 1.5 ml. In order to remove all silica residuals, the samples were centrifuged at 8000 rpm during 1 min. The supernatant was then transferred to a column and centrifuged at 13000 rpm during 10 min to purify the DNA. The fluid was discarded, 500 μL of ultra-pure water was added to the column and centrifuged at 13000 rpm during 15 min. Then, the final step was adding 120 μL of ultra-pure water to a new tube, invert the column and centrifuge at 14000 rpm during 1 min. For the Silica Solution preparation, 6 g of Silica was dissolved in 50 ml of distilled water. The solution was incubated at room temperature wrapped in aluminium foil to avoid light during 24 hours to promote silica precipitation. The supernatant was discarded and 50 ml of distilled water was added, the solution was vortexed until the dissolution of the silica pellet. Then it was incubated at room temperature covered by aluminium foil during 5 hours to promote de silica precipitation. The supernatant was discarded (about 44 ml) and 60 μL of HCL 10 M (37%) was added.

Table S1. Summary of all Cabo Verde samples used in this study. The species, with the specimen and Morphobank codes, island (I) and locality, sample type and DNA amplification success (DNA) is given. The islands are coded as F (Fogo), M (Maio), SA (Santo Antão), SN (São Nicolau), ST (Santiago), SV (São Vicente).

**Species**	**Code**	**I**	**Locality**	**Sample**	**DNA**	**Morphobank**
H. savii	MCSNG 47910a	F	São Filipe	Museum	Yes	-
H. savii	MCSNG 47910b	F	São Filipe	Museum	Yes	-
H. savii	MZS 1399	SV	Unknown	Museum	No	M675987
H. savii	MZS 12222	SV	Monte Verde	Museum	Yes	M675986
H. savii	SNQ001	SN	Monte Gordo	Wing punch	Yes	M676428-M676509
H. savii	SNQ002	SN	Monte Gordo	Wing punch	Yes	M676428-M676509
H. savii	SNQ003	SN	Monte Gordo	Wing punch	Yes	M676428-M676509
H. savii	SNQ004	SN	Monte Gordo	Wing punch	Yes	M676428-M676509
H. savii	SNQ005	SN	Monte Gordo	Wing punch	Yes	M676428-M676509
H. savii	SNQ006	SN	Cachaço	Wing punch	Yes	M676546
H. savii	InBIO 1806_12	F	São Filipe	Acoustic	-	P3514-Doc 1
P. kuhlii	MZS 10597	SV	Unknown	Museum	Yes	M675984-M675985
P. kuhlii	InBIO 1806_09	ST	Monte Caleirão	Acoustic	-	P3514-Doc 3
P. kuhlii	InBIO 1806_21	F	São Filipe	Acoustic	-	P3514-Doc 2
M. schreibersii	MZS 12221	SA	Ribeira Grande	Museum	Yes	M675988-M675989
M. schreibersii	MZS 12514	SA	Paúl	Museum	Yes	M675990-M675991
P. austriacus	MNHN 1983-1467	M	Vila do Maio	Museum	No	-
T. nudiventris	MNHN 1986-375	M	Unknown	Museum	Yes	M676419
T. nudiventris	MNHN 1986-376	M	Unknown	Museum	Yes	M676420
T. nudiventris	MNHN 1986-377	ST	Pedra Badejo	Museum	No	M676249/M676418
T. nudiventris	MNHN 1986-378	ST	Pedra Badejo	Museum	Yes	M676421
T. nudiventris	MNHN 1986-379	ST	Pedra Badejo	Museum	No	M676422
T. nudiventris	MNHN 1983-2229	ST	Trindade	Museum	Yes	M676250/M676417
T. nudiventris	InBIO 1806_11	F	São Filipe	Acoustic	-	P3514-Doc 4
T. nudiventris	STM1	ST	Calabaceira	Pellet	-	-
T. nudiventris	FM10	F	São Filipe	Pellet	-	-

Table S2. Summary of all primers used in this study. Primers are identified by gene with the sequence, species in which they were used, and the reference.

Gene	Primers	Sequence (5'-3')	Species	Reference
cyt b	F_Hs	CCTTGCAATACACTATACATCAGAC	*H. savii*	This study
cyt b	R_Hs	CCCATAGTAAAGGCCCCGTC	*H. savii*	This study
cyt b	F_PPk_m	CGGATCCCTACTGGGCATTT	*P. kuhlii*	This study
cyt b	R_PPk_m	TCCTACGTGCAGGTATAAGCA	*P. kuhlii*	This study
cyt b	F_PPh	ACGCAATTCTACGATCAATCCCTA	*P. kuhlii*	This study
cyt b	R_PPh	AACTGGCTGTCCTCCAATTCAT	*P. kuhlii*	This study
cyt b	F_PLar_teSP	GCCATACACTACACATCAGACAC	*P. austriacus*	This study
cyt b	R_PLar_teSP	GATCCGTAGTAAAGGCCTCGG	*P. austriacus*	This study
cyt b	F_PLar_teCI	AGCACTACAAATCTTAACAGGACTT	*P. austriacus*	This study
cyt b	R_PLar_teCI	TGGAGGCTCCGTTAGCATGA	*P. austriacus*	This study
cyt b	F_PLar_teTR	AGCACTACAAATCTTAACAGGACTT	*P. austriacus*	This study
cyt b	R_PLar_teTR	GGAGGCTCCGTTAGCATGAA	*P. austriacus*	This study
cyt b	F_PLma	ATCATGATGAAACTTTGGATCTCTC	*P. austriacus*	This study
cyt b	R_PLma	TCTCGGCAAATATGGGTGACA	*P. austriacus*	This study
cyt b	F_PLas	CCTTTTGAGGGGCAACCGTA	*P. austriacus*	This study
cyt b	R_PLas	GAGTTAGCGTGGCCTTGTCT	*P. austriacus*	This study
cyt b	F_Msc_ma1	TCCAAAGACTCAAGGAAAGAGCA	*M. schreibersii*	This study
cyt b	R_Msc_ma1	ACATGCTACATATTCATGGGGA	*M. schreibersii*	This study
cyt b	F_Msc_ma2	TCCTGCTCTTCGCTGTAATAG	*M. schreibersii*	This study
cyt b	R_Msc_ma2	AGAATCGGGTAAGGGTTGCT	*M. schreibersii*	This study
cyt b	F_Tn	ACGCAAATGGGGCTTCCATA	*T. nudiventris*	This study
cyt b	R_Tn	CGGTTGCGCCTCAAAAAGAT	*T. nudiventris*	This study
RAG2	RAG2-F1	GGCYGGCCCAARAGATCCTG	*H. savii*	Baker *et al.*, 2000
RAG2	RAG2-R2	GRAAGGATTTCTTGGCAGGAGT	*H. savii*	Baker *et al.*, 2000
RAG2	RAG2-R1	AACYTGYTTATTGTCTCCTGGTATGC	*H. savii*	Baker *et al.*, 2000
RAG2	RAG2-F1int	GRACAGTCGAGGGAARAGCATGG	*H. savii*	Baker *et al.*, 2000
cyt b	CB1	CCATCCAACATCTCAGCATGATGAAA	*H. savii*	Kocher *et al.*, 1989 (short)
cyt b	cytb2	CCCTCAGAATGATATTTGTCCTCA	*H. savii*	Kocher *et al.*, 1989 (short)
cyt b	cb2F	TGAGGACAAATATCATTCTGAGGG	*H. savii*	Palumbi *et al.*, 2002
cyt b	CB3H	GGCAAATAGGAARTATCATTC	*H. savii*	Palumbi *et al.*, 2002
cyt b	Molcit-F	AATGACATGAAAAATCACCGTTGT	*M. schreibersii*	Ibáñez *et al.*, 2006
cyt b	MVZ-16	AAATAGGAARTATCAYTCTGGTTTRAT	*M. schreibersii*	In Ibáñez *et al.*, 2006 (Smith & Patton 1993)

Table S3. Summary of GenBank sequences used in this study. The species name, marker, country of origin and corresponding reference of each sequence is also given.

Species	Gene	Code	Country	Reference
H. savii	*cyt b*	MT821912	Cabo Verde, São Nicolau	This study (SN01)
H. savii	*cyt b*	MT821913	Cabo Verde, São Nicolau	This study (SN02)
H. savii	*cyt b*	MT821914	Cabo Verde, São Nicolau	This study (SN04)
H. savii	*cyt b*	MT821915	Cabo Verde, São Nicolau	This study (SN06)
H. savii	*cyt b*	MT821916	Cabo Verde, São Nicolau	This study (SN03)
H. savii	*cyt b*	MT821917	Cabo Verde, São Nicolau	This study (SN05)
H. savii	*cyt b*	MT821918	Cabo Verde, São Vicente	This study (MZS12222)
H. savii	*cyt b*	MT821919	Cabo Verde, São Vicente	This study (MZS10597)
H. savii	*cyt b*	MT821920	Cabo Verde, Fogo	This study (MCSNG47910B)
H. savii	*cyt b*	MT821921	Cabo Verde, Fogo	This study (MCSNG47910A)
H. savii	*RAG2*	MT821922	Cabo Verde, São Nicolau	This study (SN01)
H. savii	*RAG2*	MT821923	Cabo Verde, São Nicolau	This study (SN02)
H. savii	*RAG2*	MT821924	Cabo Verde, São Nicolau	This study (SN03)
H. savii	*RAG2*	MT821925	Cabo Verde, São Nicolau	This study (SN04)
H. savii	*RAG2*	MT821926	Cabo Verde, São Nicolau	This study (SN05)
T. nudiventris	*cyt b*	MT821906	Cabo Verde, Maio	This study (MNHN1986_375)
T. nudiventris	*cyt b*	MT821907	Cabo Verde, Maio	This study (MNHN1986_376)
T. nudiventris	*cyt b*	MT821908	Cabo Verde, Santiago	This study (MNHN1983_2229)
T. nudiventris	*cyt b*	MT821909	Cabo Verde, Santiago	This study (MNHN1986_378)
T. nudiventris	*cyt b*	MT821910	Cabo Verde, Santiago	This study (STM1)
T. nudiventris	*cyt b*	MT821911	Cabo Verde, Fogo	This study (FM10)
M. schreibersii	*cyt b*	MT821927	Cabo Verde, Santo Antão	This study (MZS12221)
M. schreibersii	*cyt b*	MT821928	Cabo Verde, Santo Antão	This study (MZS12514)
H. savii	*cyt b*	KX375197	Cyprus	Benda *et al*., 2016
H. savii	*cyt b*	KX375196	Montenegro	Benda *et al*., 2016
H. savii	*cyt b*	KX375195	Greece	Benda *et al*., 2016
H. savii	*cyt b*	KX375194	Syria	Benda *et al*., 2016
H. savii	*cyt b*	DQ120866	Greece	Ibáñez *et al*., 2006
H. savii	*cyt b*	DQ120865	Southern Iberia	Ibáñez *et al*., 2006
H. savii	*cyt b*	DQ120864	Southern Iberia	Ibáñez *et al*., 2006
H. savii	*cyt b*	DQ120863	Southern Iberia	Ibáñez *et al*., 2006
H. savii	*cyt b*	DQ120862	Southern Iberia	Ibáñez *et al*., 2006
H. savii	*cyt b*	DQ120861	Southern Iberia	Ibáñez *et al*., 2006
H. savii	*cyt b*	DQ120860	Southern Iberia	Ibáñez *et al*., 2006
H. savii	*cyt b*	DQ120859	Northern Iberia	Ibáñez *et al*., 2006
H. savii	*cyt b*	DQ120858	Northern Iberia	Ibáñez *et al*., 2006
H. savii	*cyt b*	DQ120857	Northern Iberia	Ibáñez *et al*., 2006
H. savii	*cyt b*	KF218377	Turkey	Çoraman *et al*., 2013
H. savii	*cyt b*	KF218376	Turkey	Çoraman *et al*., 2013
H. savii	*cyt b*	KF218375	Iran	Çoraman *et al*., 2013
H. savii	*cyt b*	AJ504450	Switzerland	Stadelmann *et al*., 2004
H. savii	*cyt b*	AJ426626	Canary Islands, El Hierro	Pestano *et al*., 2002
H. savii	*cyt b*	AJ426625	Canary Islands, La Gomera	Pestano *et al*., 2002
H. savii	*cyt b*	AJ426624	Canary Islands, La Gomera	Pestano *et al*., 2002
H. savii	*cyt b*	AJ426623	Canary Islands, Tenerife	Pestano *et al*., 2002
H. savii	*cyt b*	AJ426622	Canary Islands, Gran Canaria	Pestano *et al*., 2002
H. savii	*cyt b*	AJ426621	Canary Islands	Pestano *et al*., 2002
H. savii	*cyt b*	AJ426620	Spain	Pestano *et al*., 2002
H. sp. C1	*cyt b*	EU360677	Morocco	García-Mudarra *et al*., 2009
H. sp. C2	*cyt b*	EU360678	Morocco	García-Mudarra *et al*., 2009
H. sp. C4	*cyt b*	EU360679	Morocco	García-Mudarra *et al*., 2009
H. savii	*RAG2*	EU360607	Northern Iberia	García-Mudarra *et al*., 2009

H. savii	*RAG2*	DQ120827	Switzerland	Ibáñez *et al.*, 2006
H. savii	*RAG2*	DQ120826	Northern Iberia	Ibáñez *et al.*, 2006
H. savii	*RAG2*	DQ120825	Southern Iberia	Ibáñez *et al.*, 2006
H. savii	*RAG2*	DQ120824	Southern Iberia	Ibáñez *et al.*, 2006
H. savii	*RAG2*	DQ120823	Southern Iberia	Ibáñez *et al.*, 2006
H. savii	*RAG2*	HM561667	Switzerland	Roehrs *et al.*, 2010
H. cadornae	*RAG2*	MH753143	Vietnam	Goerfoel & Csorba, 2018
H. cadornae	*RAG2*	DQ318882	Unknown	Ibáñez *et al*. unpublished
H. cadornae	*RAG2*	DQ120828	Unknown	Ibáñez *et al.*, 2006
H. cadornae	*RAG2*	GU328061	Laos	Lack *et al.*, 2010
H. pulveratus	*RAG2*	MH753145	Laos	Goerfoel & Csorba, 2018
H. sp. R1	*RAG2*	EU360606	Morocco	García-Mudarra *et al.*, 2009
H. dolichodon	*RAG2*	MH753144	Cambodia	Goerfoel & Csorba, 2018
T. nudiventris	*cyt b*	KF218430	Turkey	Çoraman *et al.*, 2013
T. nudiventris	*cyt b*	KF218429	Syria	Çoraman *et al.*, 2013
T. nudiventris	*cyt b*	HQ693713	Iran	Ruedi *et al.*, 2012
T. nudiventris	*cyt b*	HQ693712	Iran	Ruedi *et al.*, 2012
T. nudiventris	*cyt b*	MN064287	Iraq	Uvizl *et al.*, 2019
T. nudiventris	*cyt b*	MN064288	Iraq	Uvizl *et al.*, 2019
T. nudiventris	*cyt b*	MN064289	Iraq	Uvizl *et al.*, 2019
T. nudiventris	*cyt b*	MN064290	Iraq	Uvizl *et al.*, 2019
T. nudiventris	*cyt b*	MN064343	Iran	Uvizl *et al.*, 2019
T. nudiventris	*cyt b*	MN064344	Syria	Uvizl *et al.*, 2019
T. nudiventris	*cyt b*	MN064345	Syria	Uvizl *et al.*, 2019
T. nudiventris	*cyt b*	MN064346	Syria	Uvizl *et al.*, 2019
T. nudiventris	*cyt b*	MN064347	Syria	Uvizl *et al.*, 2019
T. nudiventris	*cyt b*	MN064348	Oman	Uvizl *et al.*, 2019
T. nudiventris	*cyt b*	MN064349	Syria	Uvizl *et al.*, 2019
T. nudiventris	*cyt b*	MN064350	Oman	Uvizl *et al.*, 2019
T. nudiventris	*cyt b*	MN064351	Oman	Uvizl *et al.*, 2019
T. nudiventris	*cyt b*	MN064352	Oman	Uvizl *et al.*, 2019
T. nudiventris	*cyt b*	MN064353	Oman	Uvizl *et al.*, 2019
T. nudiventris	*cyt b*	MN064354	Oman	Uvizl *et al.*, 2019
T. nudiventris	*cyt b*	MN064355	Oman	Uvizl *et al.*, 2019
T. nudiventris	*cyt b*	MN064356	Oman	Uvizl *et al.*, 2019
T. nudiventris	*cyt b*	MN064357	Oman	Uvizl *et al.*, 2019
T. nudiventris	*cyt b*	MN064358	Oman	Uvizl *et al.*, 2019
T. nudiventris	*cyt b*	MN064359	Oman	Uvizl *et al.*, 2019
T. nudiventris	*cyt b*	MN064360	Oman	Uvizl *et al.*, 2019
T. nudiventris	*cyt b*	MN064361	Syria	Uvizl *et al.*, 2019
T. nudiventris	*cyt b*	MN064362	Sudan	Uvizl *et al.*, 2019
T. nudiventris	*cyt b*	MN064363	Sudan	Uvizl *et al.*, 2019
T. nudiventris	*cyt b*	MN064364	Sudan	Uvizl *et al.*, 2019
T. nudiventris	*cyt b*	MN064365	Oman	Uvizl *et al.*, 2019
T. nudiventris	*cyt b*	MN064366	Oman	Uvizl *et al.*, 2019
T. nudiventris	*cyt b*	MN064367	Oman	Uvizl *et al.*, 2019
T. nudiventris	*cyt b*	MN064384	Ghana	Uvizl *et al.*, 2019
T. perforatus	*cyt b*	KF498635	Saudi Arabia	Memish *et al.*, 2013
T.	*cyt b*	JQ956444	Central African Republic	Maganga *et al.*, 2014
M. schreibersii	*cyt b*	FJ028649	Turkey	Furman *et al.*, 2009
M. schreibersii	*cyt b*	FJ028648	Turkey	Furman *et al.*, 2009
M. schreibersii	*cyt b*	FJ028647	Turkey	Furman *et al.*, 2009
M. schreibersii	*cyt b*	FJ028646	Turkey	Furman *et al.*, 2009
M. schreibersii	*cyt b*	FJ028631	Turkey/Georgia	Furman *et al.*, 2009
M. schreibersii	*cyt b*	FJ028630	Turkey	Furman *et al.*, 2009
M. schreibersii	*cyt b*	FJ028629	Turkey	Furman *et al.*, 2009

M. schreibersii	*cyt b*	FJ028628	Turkey	Furman *et al.*, 2009
M. schreibersii	*cyt b*	FJ028627	Turkey	Furman *et al.*, 2009
M. schreibersii	*cyt b*	FJ028626	Georgia	Furman *et al.*, 2009
M. schreibersii	*cyt b*	FJ028625	Turkey	Furman *et al.*, 2009
M. schreibersii	*cyt b*	FJ028624	Turkey	Furman *et al.*, 2009
M. schreibersii	*cyt b*	FJ028623	Turkey	Furman *et al.*, 2009
M. schreibersii	*cyt b*	FJ028622	Turkey	Furman *et al.*, 2009
M. schreibersii	*cyt b*	FJ028621	Turkey	Furman *et al.*, 2009
M. schreibersii	*cyt b*	FJ028620	Turkey	Furman *et al.*, 2009
M. schreibersii	*cyt b*	FJ028619	Turkey	Furman *et al.*, 2009
M. schreibersii	*cyt b*	FJ028618	Turkey	Furman *et al.*, 2009
M. schreibersii	*cyt b*	FJ028617	Turkey	Furman *et al.*, 2009
M. schreibersii	*cyt b*	FJ028616	Turkey	Furman *et al.*, 2009
M. schreibersii	*cyt b*	FJ028615	Turkey	Furman *et al.*, 2009
M. schreibersii	*cyt b*	FJ028614	Turkey	Furman *et al.*, 2009
M. schreibersii	*cyt b*	FJ028613	Georgia	Furman *et al.*, 2009
M. schreibersii	*cyt b*	FJ028612	Turkey	Furman *et al.*, 2009
M. schreibersii	*cyt b*	FJ028611	Turkey	Furman *et al.*, 2009
M. schreibersii	*cyt b*	FJ028610	Turkey	Furman *et al.*, 2009
M. schreibersii	*cyt b*	FJ028609	Turkey	Furman *et al.*, 2009
M. schreibersii	*cyt b*	FJ028608	Turkey	Furman *et al.*, 2009
M. schreibersii	*cyt b*	EU332392	Bulgaria_Greece_Turkey	Bilgin *et al.*, 2008
M. schreibersii	*cyt b*	EU332391	Bulgaria_Greece_Turkey	Bilgin *et al.*, 2008
M. schreibersii	*cyt b*	EU332390	Bulgaria_Greece_Turkey	Bilgin *et al.*, 2008
M. schreibersii	*cyt b*	EU332389	Bulgaria_Greece_Turkey	Bilgin *et al.*, 2008
M. schreibersii	*cyt b*	EU332388	Bulgaria_Greece_Turkey	Bilgin *et al.*, 2008
M. schreibersii	*cyt b*	EU332387	Bulgaria_Greece_Turkey	Bilgin *et al.*, 2008
M. schreibersii	*cyt b*	EU332386	Bulgaria_Greece_Turkey	Bilgin *et al.*, 2008
M. schreibersii	*cyt b*	EU332385	Bulgaria_Greece_Turkey	Bilgin *et al.*, 2008
M. schreibersii	*cyt b*	EU332384	Bulgaria_Greece_Turkey	Bilgin *et al.*, 2008
M. schreibersii	*cyt b*	EU332383	Bulgaria_Greece_Turkey	Bilgin *et al.*, 2008
M. schreibersii	*cyt b*	EU332382	Bulgaria_Greece_Turkey	Bilgin *et al.*, 2008
M. schreibersii	*cyt b*	EU332381	Bulgaria_Greece_Turkey	Bilgin *et al.*, 2008
M. schreibersii	*cyt b*	EU332380	Bulgaria_Greece_Turkey	Bilgin *et al.*, 2008
M. schreibersii	*cyt b*	EU332379	Bulgaria_Greece_Turkey	Bilgin *et al.*, 2008
M. schreibersii	*cyt b*	EU332378	Bulgaria_Greece_Turkey	Bilgin *et al.*, 2008
M. schreibersii	*cyt b*	EU332377	Bulgaria_Greece_Turkey	Bilgin *et al.*, 2008
M. schreibersii	*cyt b*	EU332376	Bulgaria_Greece_Turkey	Bilgin *et al.*, 2008
M. schreibersii	*cyt b*	EU332375	Bulgaria_Greece_Turkey	Bilgin *et al.*, 2008
M. schreibersii	*cyt b*	EU332374	Bulgaria_Greece_Turkey	Bilgin *et al.*, 2008
M. schreibersii	*cyt b*	EU332373	Bulgaria_Greece_Turkey	Bilgin *et al.*, 2008
M. schreibersii	*cyt b*	EU332372	Bulgaria_Greece_Turkey	Bilgin *et al.*, 2008
M. schreibersii	*cyt b*	EU332371	Bulgaria_Greece_Turkey	Bilgin *et al.*, 2008
M. schreibersii	*cyt b*	EU332370	Bulgaria_Greece_Turkey	Bilgin *et al.*, 2008
M. schreibersii	*cyt b*	EU332369	Bulgaria_Greece_Turkey	Bilgin *et al.*, 2008
M. schreibersii	*cyt b*	EU332368	Bulgaria_Greece_Turkey	Bilgin *et al.*, 2008
M. schreibersii	*cyt b*	EU332367	Bulgaria_Greece_Turkey	Bilgin *et al.*, 2008
M. schreibersii	*cyt b*	EU332366	Bulgaria_Greece_Turkey	Bilgin *et al.*, 2008
M. schreibersii	*cyt b*	EU332365	Bulgaria_Greece_Turkey	Bilgin *et al.*, 2008
M. schreibersii	*cyt b*	EU332364	Bulgaria_Greece_Turkey	Bilgin *et al.*, 2008
M. schreibersii	*cyt b*	EU332363	Bulgaria_Greece_Turkey	Bilgin *et al.*, 2008
M. schreibersii	*cyt b*	EU332362	Bulgaria_Greece_Turkey	Bilgin *et al.*, 2008
M. schreibersii	*cyt b*	EU332361	Bulgaria_Greece_Turkey	Bilgin *et al.*, 2008
M. schreibersii	*cyt b*	EU332360	Bulgaria_Greece_Turkey	Bilgin *et al.*, 2008
M. schreibersii	*cyt b*	EU332359	Bulgaria_Greece_Turkey	Bilgin *et al.*, 2008
M. schreibersii	*cyt b*	EU332358	Bulgaria_Greece_Turkey	Bilgin *et al.*, 2008

M. schreibersii	*cyt b*	EU332357	Bulgaria_Greece_Turkey	Bilgin *et al*., 2008
M. schreibersii	*cyt b*	EU332356	Bulgaria_Greece_Turkey	Bilgin *et al*., 2008
M. schreibersii	*cyt b*	EU332355	Bulgaria_Greece_Turkey	Bilgin *et al*., 2008
M. schreibersii	*cyt b*	AY923073	Turkey	Bilgin *et al*., 2006
M. schreibersii	*cyt b*	AY923072	Turkey	Bilgin *et al*., 2006
M. schreibersii	*cyt b*	AY923071	Turkey	Bilgin *et al*., 2006
M. schreibersii	*cyt b*	AY923069	Greece	Bilgin *et al*., 2006
M. schreibersii	*cyt b*	AY923068	Greece	Bilgin *et al*., 2006
M. schreibersii	*cyt b*	AY923067	Bulgaria	Bilgin *et al*., 2006
M. schreibersii	*cyt b*	AY923065	Bulgaria	Bilgin *et al*., 2006
M. schreibersii	*cyt b*	AY923064	Turkey	Bilgin *et al*., 2006
M. schreibersii	*cyt b*	HM047792	Turkey	Furman *et al*., 2010
M. schreibersii	*cyt b*	HM047791	Turkey	Furman *et al*., 2010
M. schreibersii	*cyt b*	HM047790	Turkey	Furman *et al*., 2010
M. schreibersii	*cyt b*	HM047789	Turkey	Furman *et al*., 2010
M. schreibersii	*cyt b*	HM047788	Turkey	Furman *et al*., 2010
M. schreibersii	*cyt b*	HM044090	Turkey	Furman *et al*., 2010
M. schreibersii	*cyt b*	HM044089	Turkey	Furman *et al*., 2010
M. schreibersii	*cyt b*	HM044088	Turkey	Furman *et al*., 2010
M. schreibersii	*cyt b*	HM044087	Turkey	Furman *et al*., 2010
M. schreibersii	*cyt b*	HM044086	Turkey	Furman *et al*., 2010
M. schreibersii	*cyt b*	HM044085	Turkey	Furman *et al*., 2010
M. schreibersii	*cyt b*	HM044084	Turkey	Furman *et al*., 2010
M. schreibersii	*cyt b*	HM044083	Turkey	Furman *et al*., 2010
M. schreibersii	*cyt b*	HM044082	Turkey	Furman *et al*., 2010
M. schreibersii	*cyt b*	HM044081	Turkey	Furman *et al*., 2010
M. schreibersii	*cyt b*	HM044080	Turkey	Furman *et al*., 2010
M. schreibersii	*cyt b*	HM044079	Turkey	Furman *et al*., 2010
M. schreibersii	*cyt b*	HM044078	Turkey	Furman *et al*., 2010
M. schreibersii	*cyt b*	HM044077	Turkey	Furman *et al*., 2010
M. schreibersii	*cyt b*	HM044076	Turkey	Furman *et al*., 2010
M. schreibersii	*cyt b*	HM044075	Turkey	Furman *et al*., 2010
M. schreibersii	*cyt b*	HM044074	Turkey	Furman *et al*., 2010
M. schreibersii	*cyt b*	HM044073	Turkey	Furman *et al*., 2010
M. schreibersii	*cyt b*	HM044072	Turkey	Furman *et al*., 2010
M. schreibersii	*cyt b*	HM044071	Turkey	Furman *et al*., 2010
M. schreibersii	*cyt b*	KX008566	Unknown	Bilgin *et al*., 2016
M. schreibersii	*cyt b*	KX008565	Unknown	Bilgin *et al*., 2016
M. schreibersii	*cyt b*	KX008564	Unknown	Bilgin *et al*., 2016
M. schreibersii	*cyt b*	KX008563	Unknown	Bilgin *et al*., 2016
M. schreibersii	*cyt b*	KX008562	Balkans	Bilgin *et al*., 2016
M. schreibersii	*cyt b*	KX008561	Anatolia	Bilgin *et al*., 2016
M. schreibersii	*cyt b*	KX008560	Anatolia	Bilgin *et al*., 2016
M. schreibersii	*cyt b*	KX008559	Balkans	Bilgin *et al*., 2016
M. schreibersii	*cyt b*	KX008558	Anatolia	Bilgin *et al*., 2016
M. schreibersii	*cyt b*	KX008557	Anatolia	Bilgin *et al*., 2016
M. schreibersii	*cyt b*	KX008556	Anatolia	Bilgin *et al*., 2016
M. schreibersii	*cyt b*	KX008555	Anatolia	Bilgin *et al*., 2016
M. schreibersii	*cyt b*	KX008554	Black Sea	Bilgin *et al*., 2016
M. schreibersii	*cyt b*	KX008553	Anatolia	Bilgin *et al*., 2016
M. schreibersii	*cyt b*	KX008552	Black Sea	Bilgin *et al*., 2016
M. schreibersii	*cyt b*	KX008551	Anatolia	Bilgin *et al*., 2016
M. schreibersii	*cyt b*	KX008550	North Africa	Bilgin *et al*., 2016
M. schreibersii	*cyt b*	KX008549	Iberia	Bilgin *et al*., 2016
M. schreibersii	*cyt b*	KX008548	Balkans	Bilgin *et al*., 2016
M. schreibersii	*cyt b*	KX008547	Russia	Bilgin *et al*., 2016

M. schreibersii	*cyt b*	KX008546	Balkans	Bilgin *et al*., 2016
M. schreibersii	*cyt b*	KX008545	Balkans	Bilgin *et al*., 2016
M. schreibersii	*cyt b*	KX008544	Balkans	Bilgin *et al*., 2016
M. schreibersii	*cyt b*	KX008543	Balkans_Slovakia	Bilgin *et al*., 2016
M. schreibersii	*cyt b*	KX008542	Balkans	Bilgin *et al*., 2016
M. schreibersii	*cyt b*	KX008541	Morocco	Bilgin *et al*., 2016
M. schreibersii	*cyt b*	KX008540	North Africa	Bilgin *et al*., 2016
M. schreibersii	*cyt b*	KX008539	North Africa	Bilgin *et al*., 2016
M. schreibersii	*cyt b*	KX008538	Morocco	Bilgin *et al*., 2016
M. schreibersii	*cyt b*	KX008537	Unknown	Bilgin *et al*., 2016
M. schreibersii	*cyt b*	KX008536	Lebanon	Bilgin *et al*., 2016
M. schreibersii	*cyt b*	KX008535	Balkans	Bilgin *et al*., 2016
M. schreibersii	*cyt b*	KX008534	Unknown	Bilgin *et al*., 2016
M. schreibersii	*cyt b*	KX008533	Balkans	Bilgin *et al*., 2016
M. schreibersii	*cyt b*	KX008532	France	Bilgin *et al*., 2016
M. schreibersii	*cyt b*	KX008531	France	Bilgin *et al*., 2016
M. schreibersii	*cyt b*	KX008530	France	Bilgin *et al*., 2016
M. schreibersii	*cyt b*	KX008529	France	Bilgin *et al*., 2016
M. schreibersii	*cyt b*	KX008528	Cyprus	Bilgin *et al*., 2016
M. schreibersii	*cyt b*	KX008527	Albania	Bilgin *et al*., 2016
M. schreibersii	*cyt b*	KX008526	Balkans	Bilgin *et al*., 2016
M. schreibersii	*cyt b*	KX008525	Balkans	Bilgin *et al*., 2016
M. schreibersii	*cyt b*	KX008524	Balkans	Bilgin *et al*., 2016
M. schreibersii	*cyt b*	KX008523	Albania	Bilgin *et al*., 2016
M. schreibersii	*cyt b*	KX008522	Balkans	Bilgin *et al*., 2016
M. schreibersii	*cyt b*	KJ535860	Spain	Puechmaille *et al*., 2014
M. schreibersii	*cyt b*	KJ535858	Romania	Puechmaille *et al*., 2014
M. schreibersii	*cyt b*	KJ535852	Lebanon	Puechmaille *et al*., 2014
M. schreibersii	*cyt b*	KJ535850	Slovenia	Puechmaille *et al*., 2014
M. schreibersii	*cyt b*	KJ535849	Romania	Puechmaille *et al*., 2014
M. schreibersii	*cyt b*	KJ535845	France	Puechmaille *et al*., 2014
M. schreibersii	*cyt b*	KJ535842	Tunisia	Puechmaille *et al*., 2014
M. schreibersii	*cyt b*	KJ535841	Lebanon	Puechmaille *et al*., 2014
M. schreibersii	*cyt b*	KJ535839	Slovenia	Puechmaille *et al*., 2014
M. schreibersii	*cyt b*	KJ535837	Russia	Puechmaille *et al*., 2014
M. schreibersii	*cyt b*	KJ535836	Romania	Puechmaille *et al*., 2014
M. schreibersii	*cyt b*	KJ535835	Romania	Puechmaille *et al*., 2014
M. schreibersii	*cyt b*	KJ535833	Croatia	Puechmaille *et al*., 2014
M. schreibersii	*cyt b*	KJ535832	Croatia	Puechmaille *et al*., 2014
M. schreibersii	*cyt b*	KJ535830	Albania	Puechmaille *et al*., 2014
M. schreibersii	*cyt b*	KJ535829	Morocco	Puechmaille *et al*., 2014
